# Sleep and light ecologies of parents with young infants: descriptive findings from the SUNSHINE project

**DOI:** 10.1038/s44323-026-00098-w

**Published:** 2026-07-27

**Authors:** Vaida Verhoef, Emma Visser, Karin Smolders, Niki Antypa, Yvonne de Kort

**Affiliations:** 1 Human-Technology Interaction, Department of Industrial Engineering and Innovation Sciences, Eindhoven University of Technology, Eindhoven, The Netherlands; 2 Department of Clinical Psychology, Faculty of Social Sciences, Leiden University, Leiden, The Netherlands

**Keywords:** Health care, Neuroscience, Physiology, Psychology, Psychology

## Abstract

The interplay between sleep, light exposure, and circadian rhythms is critical for health, yet it remains inadequately described in the everyday lives of parents with young infants. Prior work has focused mainly on mothers, the early postpartum period, and short-term assessments, leaving a limited understanding of everyday parental ecologies. To address these gaps, we conducted a 2-week observation study in both mothers and fathers of infants aged 3–9 months, simultaneously measuring sleep and light exposure using wearable actigraphy and light sensors alongside sleep diaries. Our descriptive approach aimed to characterize real-life patterns of sleep and light, quantify their variability across nights and individuals, and explore differences by infant age, parental role, and contextual factors. We found that while sleep duration was generally adequate, nights were frequently fragmented, with multiple awakenings and substantial variability across individuals and nights. Light exposure during waking hours was predominantly dim, and brief exposures to light during sleep episodes were common. Seasonal variation, infant sleep location, and caregiving roles may have shaped sleep-light patterns. These findings highlight the complexity and heterogeneity of sleep and light ecologies in early parenthood, and underscore the value of wearable monitoring and descriptive profiling for informing circadian-aware interventions.

## Introduction

In the midst of physiological and emotional changes, the arrival of an infant brings about significant shifts in family dynamics, with disrupted sleep patterns emerging as one of the most critical challenges. The postpartum period—reflecting the span of time after childbirth—has predominantly been studied among primiparous mothers, in relation to risks and prevalence of depression^1,2^. Vulnerability to mood disorders has been linked to sleep disruptions occasioned by infant care (particularly nocturnal awakenings and feeding^3–5^), although postpartum depression has also been frequently attributed to major hormonal fluctuations^6^. These sleep disruptions can, in turn, have downstream consequences on psychological health^7^, breastfeeding satisfaction^8,9^, parental self-efficacy^3^, parent-child bonding^10^, cognitive functioning^11,12^, fatigue^6,13^, and overall quality of life^14^. Together, these wide-ranging consequences underscore the central role of sleep in shaping parental well-being during the postpartum period.

While the majority of studies on sleep disturbances during the postpartum period have focused on (primiparous) mothers^1,15,16^, fathers also experience changes due to the arrival of the baby: sleep disruptions and their consequences^4,11,15,17,18^. For both parents, the postpartum period is associated with short sleep, lower sleep efficiency, and increased occurrence and duration of nighttime awakenings^4,15,19^. Multiple studies have noted significant changes in sleep (especially self-rated sleep quality and sleep durations) before and after childbirth^18,20^. Yet, only a few studies have extended their focus beyond sleep deprivation, onto increased sleep fragmentation, decreased sleep efficiency^15^, or disturbed sleep–wake regulation and their repercussions on (mental) health^10,21–23^.

Sleep regulation is closely tied to circadian rhythms, yet research on circadian health in new parents is still emerging. Evidence suggests a change in the circadian rhythms of mothers (as indexed by melatonin secretion, as well as actimetry-derived rest-activity rhythm)^24,25^, with a potential link to risk of postpartum depression^26,27^. According to a systematic review by Gallaher et al., 18 studies (at the time) investigated circadian rhythms in mothers after childbirth^28^. Yet, only one study included mothers after the first few months of postpartum^26^. However, sleep disturbances and shifts in the circadian rhythm appear to extend well beyond the early postpartum months^13,29^. At 1 year postpartum, sleep disturbance and fatigue are still reported by parents^13,29,30^. Additionally, several investigations have used multi-day actigraphy to examine the detailed dynamics of parental sleep^2,15,28,31^. However, these studies exhibit inconsistencies in their protocols, rarely conduct day-to-day analyses, only include a few fathers, and primarily focus on the first few months postpartum, leaving gaps in understanding how sleep–wake patterns and sleep disruptions vary across days and between parents.

Light is the primary zeitgeber (time cue) of circadian rhythms and exerts a strong influence on sleep–wake regulation, mood, and overall health^32–34^. Despite its recognized importance for health beyond vision^33,34^, light exposure has rarely been investigated among parents of young infants. Of the studies reviewed by Gallaher et al. focusing on circadian rhythm in mothers^28^, only two mentioned light (interventions for depression prevention^35^ or sleep consolidation within this population^36^), despite evidence that both bright light during the day and reduced light exposure at night are critical for circadian alignment and mood regulation^37,38^.

In fact, most studies on light in relation to the postpartum period (e.g., targeting sleep, circadian rhythm, and/or mood disorders) have focused on exploring light interventions (such as bright light therapy or blue light blocking glasses), which resulted in mixed or inconclusive results^35,36,39–43^. Yet, without systematic descriptions of naturally occurring light exposure in parents with young infants, it remains unclear whether existing interventions address genuine deficits or target the most relevant dimensions of light exposure. In fact, to our knowledge, only four studies have monitored naturally occurring variations in light exposure (including natural and electrical light) during the postpartum period^16,44–46^. The studies performed by Wang et al.^45^ and Goyal et al.^46^ only report on 24h-mesors of illuminance and photoperiods categorized as short vs. long days, respectively. Lee et al.^16^ monitored light exposure at the wrist of twenty mothers and reported a mean illuminance of 73 lux (over a 12-h waking period), with light ranging from 3.4 to 218 lux. Tsai et al.^44^ investigated realistic light exposure in more detail by monitoring the light exposure of 24 mother-infant pairs over a week. Illuminance was recorded at the wrist of mothers and at the ankle of infants (less than 3 months old) and then averaged over days. The authors noted low illumination levels during the wake episode, with an average of 71.13% (SD = 11.58) of the day (between 06:00 and 21:59) spent by mothers in an environment below 50 lux (measured at wrist level). In comparison, the average time spent in a bright environment (>1000 lux) was 54 min (SD = 39 min). During nighttime, mothers appeared to spend on average 96.6% (SD = 1.08) of their time in light environments below 15 lux, with, however, maximum recording levels reaching 237 ± 121 lux (recorded at bedside). According to these studies, daytime and nighttime light exposure patterns of mothers with young infants appear to diverge markedly from light thresholds mentioned in the literature (above 250 lux during daytime and below 1 lux during nighttime)^37,38^. Unfortunately, the study by Tsai et al. had some methodological limitations, including monitoring light exposure at wrist-level, and averaging across large time windows (hampering insights into time profiles). Sensors closer to eye level (e.g., at the collar) would have given a better estimation than at wrist level (where sleeves can easily obstruct the sensor) or at bedside level. Before therapeutic light interventions can be considered, a systematic monitoring of light exposure in naturalistic settings (both during wakefulness and sleep) is needed to better understand light exposure patterns among parents of young infants, how they compare to recommendations, and how they covary with disturbances in rest-activity patterns.

In this study, we explored the light and sleep ecologies of mothers and fathers of infants aged 3–9 months. Using 2 weeks of continuous actigraphy and light monitoring in parents, we mapped real-world patterns and their variability across time, and in function to infant and parent characteristics. Rather than testing specific hypotheses, our aim was deliberately descriptive: to provide a detailed characterization of everyday sleep-light environments in early parenthood and identify sources of variation that may inform future mechanistic and intervention research.

## Results

### Sample Description

Tables 1 and 2 present characteristics of our sample, including information about the parents as well as their children. A total of 18 parents participated in the study. Parents were between 28 and 37 years of age (*M* = 32.6 years), and the sample included both mothers (*n* = 12) and fathers (*n* = 6). Twelve of the parents were primiparous parents, while 5 had another child (2–6 years old). Employment status varied, with most parents working full-time (*n* = 8) or part-time (*n* = 5), while others were on parental leave or not working (*n* = 3). One parent did not provide demographic information and was only included in group-level analyses. The children ranged in age from 13 to 43 weeks at the time of assessment. Most infants (*n* = 10) slept in the parents’ room, while some slept in their own room (*n* = 6). Data collection was carried out in two seasons. During summer (July–September), the duration of the natural photoperiod ranged from 13.29 to 17.29 h, while in winter (November–December) it was substantially shorter, between 9.18 and 10.65 h.

**Table 1:** Parent characteristics

Parent	Season	Gender	Work	ESS	PSQI	MSFsc	PSS	EPDS
F-201	Summer	Female	Part-Time Employment	3	11	03:00:00	73	15
M-221	Summer	Male	Full-Time Employment	15	16	03:41:47	61	15
F-231-C1	Summer	Female	Full-Time Employment	5	5	03:17:08	54	2
M-232-C1	Summer	Male	Part-Time Employment	0	4	03:10:10	52	4
F-241-C2	Summer	Female	Full-Time Employment	2	9	02:00:00	62	11
M-242-C2	Summer	Male	Full-Time Employment	5	11	02:30:00	62	4
M-251	Summer	Male	Full-Time Employment	10	6	03:11:25	62	6
F-261	Summer	Female	Part-Time Employment	5	3	03:02:08	60	7
F-271	Summer	Female	Part-Time Employment	5	9	03:45:00	68	6
F-281	Winter	Female	Full-Time Employment	13	5	01:51:57	69	18
F-291	Winter	Female	Part-Time Employment	13	8	02:28:23	59	9
F-301-C3	Winter	Female	Not working / Parental Leave	9	10	03:11:47	69	10
M-302-C3	Winter	Male	Full-Time Employment	2	8	03:20:00	64	12
F-311-C4	Winter	Female	Not working / Parental Leave	6	9	02:30:00	72	8
M-312-C4	Winter	Male	Full-Time Employment	10	6	02:40:42	56	7
M-321	Winter	Male	Part-Time Employment	4	9	03:05:53	64	10
F-331	Winter	Female	NA	NA	NA	NA	NA	NA
F-341	Winter	Female	Not working / Parental Leave	15	10	05:09:38	70	12

*ESS* Epworth Sleepiness Scale, *PSQI* Pittsburgh Sleep Quality Index, *MSFsc* mid-sleep on free days (sleep-debt corrected), *PSS* Parental Stress Scale, *EPDS* Edinburgh Postnatal Depression Scale, *NA* not available.

**Table 2:** Infant characteristics and sleep environment

Parent	Baby age (weeks)	Baby age (categorical)	Day care	Bedroom	Bed	Sleep duration^a^	Awakenings^a^
F-201	30	>6 months	2 days	Parent room	Parents’ bed	10:00:00	2
M-221	34	>6 months	3 days	Parent room	Crib	08:45:00	5
F-231-C1	14	<6 months	4 days	Own room	Own bed	10:15:00	1
M-232-C1	14	<6 months	3 days	Own room	Own bed	10:00:00	0
F-241-C2	21	<6 months	0 days	Parent room	Crib	09:30:00	3
M-242-C2	21	<6 months	0 days	Parent room	Crib	09:30:00	2
M-251	13	<6 months	3 days	Parent room	Crib	09:45:00	2
F-261	37	>6 months	3 days	Own room	Crib	10:30:00	2
F-271	29	>6 months	4 days	parent room	Crib	11:00:00	0
F-281	43	>6 months	3 days	Own room	Crib	10:40:00	1
F-291	25	>6 months	4 days	Parent room	Crib	10:30:00	3
F-301-C3	24	<6 months	4 days	Parent room	Crib	11:50:00	3
M-302-C3	24	<6 months	4 days	Parent room	Crib	09:00:00	3
F-311-C4	16	<6 months	0 days	Parent room	Co-sleeper	10:30:00	4
M-312-C4	16	<6 months	0 days	Parent room	Co-sleeper	10:00:00	4
M-321	20	<6 months	3 days	own room	Own bed	12:00:00	3
F-331	NA	NA	NA	NA	NA	NA	NA
F-341	14	<6 months	0 days	own room	Own bed	08:30:00	2

*NA* not available.

^a^refers to the infant's sleep duration and awakenings.

### Nighttime sleep: adequate duration with frequent disruptions

**Fig. 1:**
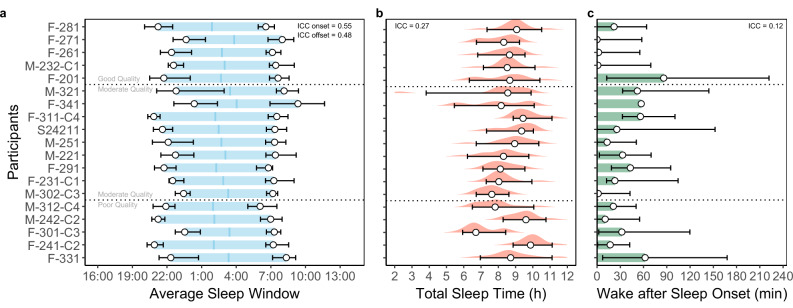
Distributions of actigraphy-derived sleep across parents. Panels show, for each parent, the median and 95% confidence interval of **a** sleep onset and sleep offset (the vertical blue line indicates the average midsleep), **b** total sleep time, and **c** wake after sleep onset (WASO). Parents are ordered (top to bottom) by their median diary-based sleep quality, with horizontal dotted lines marking group boundaries corresponding to poor (≤3), moderate (4–5), and good (≥6) ratings on the 1–7 scale. Intraclass correlation coefficients (ICC) reflect the proportion of total variance attributable to stable between-person differences.

Figure 1 and Table S1 summarize nighttime sleep in our cohort as measured using actigraphy. Parents fell asleep shortly after 21:00 (21:02 ± 00:48) and woke around 07:25 (±00:37), spending 9 h 09 m ± 1 h 09 m in bed (TIB) on average. Compared to actigraphy, diaries indicated later sleep onset (+48 min; limits of agreement [LOA] +14 to +83 min) and earlier sleep offset (−30 min; LOA −72 to +13 min), resulting in approximately 78 min less time in bed (see Table S2). Most parents were morning types (12/19; 63%), with six classified as intermediate (32%) and only one as an evening type (5%). Self-reported sleep quality using the Pittsburgh Sleep Quality Index (PSQI) indicated global scores ranging from 3 to 16, with 15/17 parents having a score of 5 or higher (mean 8.2 ± 3.2).

Nights of the parents were quite fragmented on average. Parents experienced a wake after sleep onset (WASO) of 40 ± 40 min and 2.5 ± 1.3 awakenings per night, as measured by actigraphy. The mean longest continuous sleep bout lasted 5 h 48 m ± 1 h 33 m, while the average fragmentation index was 12.8% ± 6.0%. Despite these interruptions, average total sleep time (TST) remained at 8 h 29 m ± 1 h 00 min, with high sleep efficiency (93.2% ± 5.9%).

**Fig. 2:**
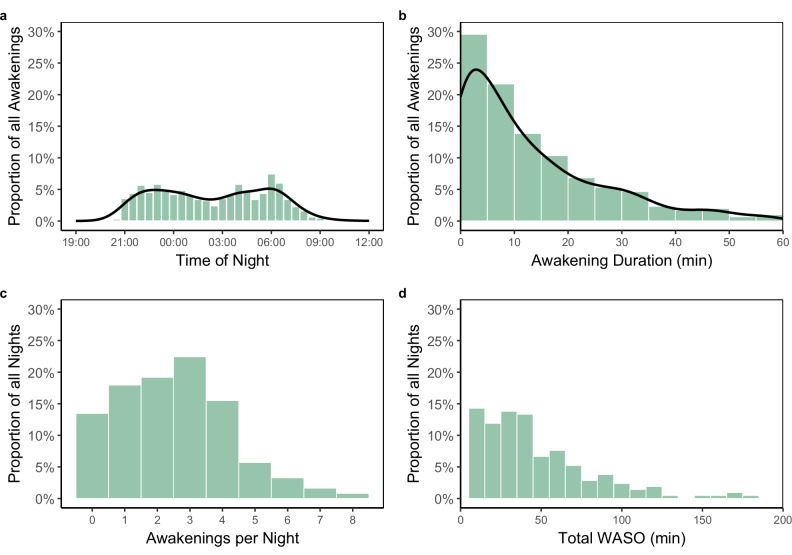
Nighttime awakening patterns measured by actigraphy. **a** Distribution of the timing of awakening onsets across the night, aggregated across all parents and nights. Bars represent the percentage of all awakenings falling within 30-min bins; the black line is a smoothed density estimate. Two peaks are evident: shortly after sleep onset and again in the early morning hours. **b** Distribution of awakening durations: histogram of individual events with a smoothed density overlay, showing that most awakenings are short (under about 10 min). **c** Distribution of the number of awakenings per night. Bars show the percentage of nights with a given count of awakenings; most nights feature two to three, and only a small proportion of the nights (13.5%) were free of awakenings. **d** Distribution of total Wake After Sleep Onset (WASO) per night, expressed as the percentage of nights in each WASO bin. Most nights involve under 50 min of cumulative wakefulness, though a subset extends well beyond 100 min (up to ~200 min).

Figure 2 provides a more detailed overview of specifically the nighttime disruptions (measured with actigraphy). Awakenings occurred throughout the night, but showed small peaks shortly after sleep onset (22:00–00:00) and again in the early morning (4:30-6:30), with relatively fewer awakenings during the middle of the night (Fig. 2a). Most awakenings were brief, typically lasting under 10 min. Awakenings longer than 45 min were rare; all but five parents experienced at least one such prolonged awakening during the 2-week period (Fig. 2b). Across nights, parents experienced between zero and eight awakenings, with three awakenings being most common and only 13.5% of nights free of nocturnal awakenings (Fig. 2c). Most nights involved under 50 min of cumulative WASO, although some extended to 150–200 min, reflecting substantial inter-night variability (Fig. 2d).

### From consolidated to fragmented: individual differences in parental sleep

**Fig. 3:**
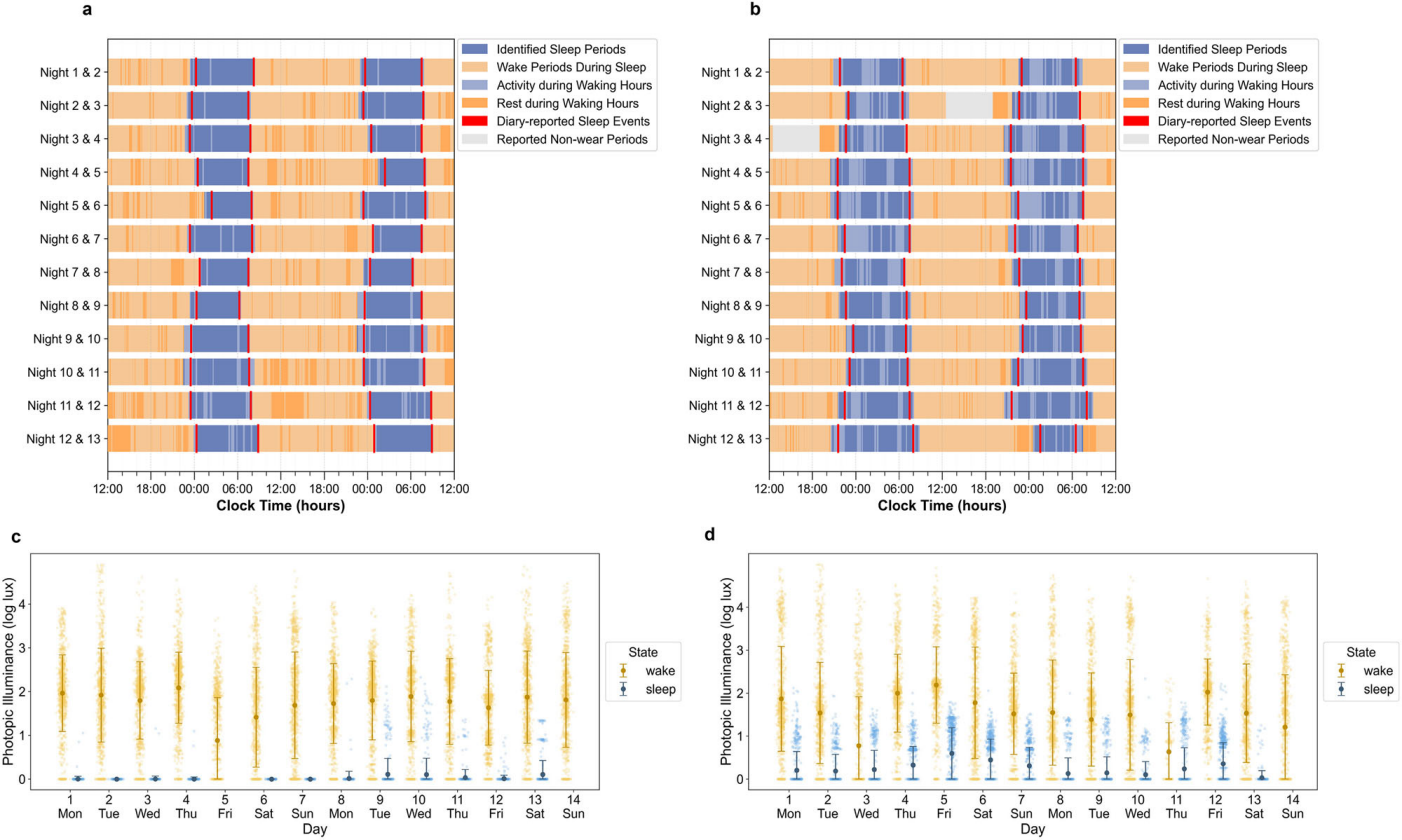
Forty-eight-hour double-plotted actigrams and associated light profiles for two exemplar parents representing the two illustrative sleep phenotypes. **a** “Pristine sleeper” (F-271) exhibits long, uninterrupted sleep episodes with minimal wake after sleep onset (WASO < 20 min and <1 awakening per night). **b** “Extremely fragmented sleeper” (F-201) shows frequent interruptions with multiple awakenings, resulting in shorter, punctuated sleep bouts (WASO > 60 min and >3 awakenings per night). **c** “Pristine sleeper” presents very few and dim light exposures during sleep episode as represented in blue dots (mean illuminance) and scatter. **d** “Extremely fragmented sleeper” shows frequent light exposures during sleep episodes, reaching above 100 lux (or 2 log lux).

Substantial between-parent variability in sleep patterns was observed in our sample. To illustrate this heterogeneity, we present two contrasting descriptive sleep profiles: the “Pristine Sleeper” and the “Extremely Fragmented Sleeper” (Fig. 3). These profiles were defined based on actigraphy-derived WASO and the number of nocturnal awakenings. The “Pristine Sleeper” profile was defined as an average WASO below 20 min and fewer than one awakening per night, whereas the “Extremely Fragmented Sleeper” profile was defined as an average WASO exceeding 60 min and more than three awakenings per night. These thresholds were selected to represent examples of relatively consolidated and highly fragmented sleep, broadly informed by normative ranges reported in previous sleep literature^47^. The labels of “Pristine Sleeper” and the “Extremely Fragmented Sleeper” were intended solely as descriptive illustrations of contrasting sleep patterns observed within the current sample and should not be interpreted as clinical classifications or distinct sleep phenotypes.

In our cohort, 3 of 19 parents (M-232-C1, F-261, F-271) met the predefined criteria for the illustrative “Pristine Sleeper” profile, while 4 of 19 (F-201, M-321, F-331, F-341) represented the opposite extreme as “Extremely Fragmented Sleepers.” The remaining 12 parents occupied intermediate positions, losing between 20 and 60 min to WASO and typically experiencing 1–3 awakenings nightly. Figure 3a illustrates the actigram of a “Pristine Sleeper” (parent F-271). Across 13 nights, sleep appears in long, consolidated blocks of 7–8 h (average of 7 h 18 m ± 1:21), with only 9 awakenings in total, of which 2 were longer than 20 min (as measured using actigraphy). This parent averaged only about 10 min of actigraphy-derived WASO and fewer than one awakening per night, yielding an efficiency of 97.8% ± 3.8% and a fragmentation index of just 5.9% ± 4.8%. In short, their sleep was nearly ideal in both duration and continuity.

By contrast, Fig. 3b depicts an “Extremely Fragmented Sleeper,” (parent F-201) whose actigrams are punctuated by frequent and prolonged periods of wakefulness. Each night contained 3.38 ± 1.94 awakenings on average, adding up to 1 h 31 m ± 1 h 06 m of WASO. These repeated awakenings broke the night into shorter bouts, with the longest uninterrupted sleep period averaging only 4 h 24 m ± 1:13. Throughout the duration of the study, this parent experienced a total of 44 awakenings, 20 of which exceeded 20 min in length. Sleep efficiency regularly fell below 80% (with a mean of 85.1% ± 10.8%), and the fragmentation index exceeded 20% (22.1% ± 10.6%). Despite spending a comparable total time in bed as the participant displayed in Fig. 3a, this parent’s sleep was persistently fractured.

### Subjective sleep quality does not reflect objective sleep

Subjective sleep quality (SSQ) was generally moderate, but variable, across the cohort. Median nightly sleep-quality ratings spanned the full 7-point scale across parents, with a cohort median of 4 (“Neither Good nor Bad Quality”). Restedness scores also varied substantially across parents, spanning the full scale (1–7), with a cohort median of 3 (“Somewhat Unrested”).

Subjective evaluations of sleep were often more positive than might be expected based on actigraphy-derived sleep metrics alone. Parents meeting the criteria for the illustrative “Pristine Sleeper” profile generally reported sleep-quality scores of 5–6 (“Somewhat Good”–“Good”) and restedness scores of 5 (“Quite Rested”), above the cohort medians. However, among parents meeting the criteria for the “Extremely Fragmented Sleeper” profile, subjective ratings were more positive than might be expected: despite experiencing over an hour of WASO on average, three of the four parents still reported sleep quality around 5 and restedness around 3, while only one parent reported poor sleep quality and restedness. This suggests that substantial sleep fragmentation can coexist with relatively positive subjective evaluations of sleep (see Fig. 1c).

The retrospective PSQI aligned somewhat better with these illustrative sleep profiles. Two of the three parents meeting the criteria for the “Pristine Sleeper” profile scored within the healthy PSQI range (<4), whereas all parents meeting the criteria for the “Extremely Fragmented Sleeper” profile scored within the poor range (9–11).

### Daytime napping behavior

Sleep disturbances at night can also lead to changes in daytime activity patterns—specifically, more frequent naps or extended periods of rest. In this cohort, subjectively reported daytime napping behavior is reported in Table 3. Although the median number of diary-reported naps per day was 0 (range: 0–2), parents reported napping on 19.4% of all observation days. When naps occurred, the average total reported nap duration was 49.9 min (SD = 54.6). There were also substantial interindividual differences. Some parents reported no naps at all during the study period (20% of the cohort). Of the 80% of parents who reported naps, some reported frequent short naps (e.g., F-241-C2), and a few reported frequent long naps (e.g., F-261).

**Table 3:** Subjectively reported naps and actigraphy-recorded rest periods

	Subjectively reported naps			Actigraphy-recorded rest periods		
Parent	Episodes/day	% days with recording	Total daily duration (min)	Episodes/day	% days with recording	Total daily duration (min)
F-201	0.0 (0–0)	0%	–	4 (1–9)	100%	226.8 (172.2)
M-221	0.0 (0–2)	21.4%	20.0 (8.7)	6.5 (0–11)	92.9%	307.8 (91.2)
F-231-C1	0.0 (0–1)	7.14%	5.0 (–)	10 (5–15)	100%	360.0 (116.4)
M-232-C1	0.0 (0–0)	0%	–	5.5 (2–13)	100%	236.4 (114.0)
F-241-C2	0.5 (0–2)	50%	14.3 (7.3)	6.5 (4–11)	100%	366.6 (184.2)
M-242-C2	0.0 (0–2)	21.4%	38.3 (18.9)	6 (0–10)	92.9%	330.6 (186.6)
M-251	0.0 (0–1)	14.3%	69.0 (29.7)	4.5 (1–8)	100%	204.0 (79.2)
F-261	0.0 (0–2)	42.9%	125.0 (106.0)	4 (1–10)	100%	218.4 (138.6)
F-271	0.0 (0–2)	28.6%	87.5 (68.0)	5 (2–11)	100%	216.0 (130.2)
F-281	0.0 (0–0)	0%	–	6 (3–11)	100%	357.0 (273.0)
F-291	0.0 (0–2)	14.3%	0.0 (14.1)	9 (6–12)	100%	406.8 (108.0)
F-301-C3	0.0 (0–2)	33.3%	50.0 (35.4)	7.5 (5–9)	100%	318.0 (65.4)
M-302-C3	0.0 (0–1)	8.33%	20.0 (–)	8 (3–14)	100%	409.8 (129.6)
F-311-C4	0.0 (0–1)	7.14%	30.0 (–)	5.5 (3–10)	100%	279.0 (87.0)
M-312-C4	0.0 (0–1)	8.33%	10.0 (–)	6.5 (1–13)	100%	304.2 (126.6)
M-321	0.0 (0–1)	21.4%	35.0 (35.0)	4.5 (1–11)	100%	173.4 (134.4)
F-331	1.0 (0–1)	61.5%	46.9 (18.7)	5 (2–11)	100%	352.2 (220.2)
F-341	0.0 (0–1)	28.6%	46.2 (49.9)	10 (6–13)	100%	400.8 (117.0)
**Group**	**0.0 (0–2)**	**19.4%**	**49.9 (54.6)**	**6 (1–15)**	**99.2%**	**309.0 (180.0)**

“Episodes per day” is depicted as median (range) and “Total daily duration” as mean (SD).

“Rest” time across daylight hours, as measured by actigraphy, is also summarized in Table 3. In contrast to the diary self-reports, actigraphy detected rest periods during the photoperiod on nearly every day of the study (99.2% of observation days). Parents had a median of 6 rest episodes per day (range: 1–15), with a mean cumulative daily rest duration of 309 min (SD = 180).

### Stable days, variable nights: sleep regularity and nightly variability

A complementary perspective on parental sleep–wake behavior is provided by measures of daily rhythm regularity. Measures of Interdaily Stability (IS = 0.45–0.78, mean ± SD = 0.66 ± 0.09), Intradaily Variability (IV = 0.27–0.54, mean ± SD = 0.38 ± 0.08), and the Sleep Regularity Index (SRI = 62.5–81.3%, mean ± SD = 72.5 ± 4.1%) suggested that most parents maintained relatively regular sleep–wake schedules (Table 4).

**Table 4:** Regular matrices of sleep and of photopic light exposure for each parent

	Sleep			Light	
Parent	SRI (%)	IV	IS	IV	IS
F-201	73.22	0.33	0.65	1.65	0.29
M-221	72.39	0.39	0.70	1.35	0.32
F-231-C1	68.51	0.45	0.67	1.34	0.20
M-232-C1	75.54	0.32	0.78	1.75	0.30
F-241-C2	68.45	0.44	0.61	1.61	0.23
M-242-C2	74.59	0.38	0.68	1.57	0.28
M-251	75.21	0.31	0.67	1.31	0.26
F-261	81.33	0.30	0.71	1.23	0.24
F-271	76.17	0.30	0.67	1.71	0.17
F-281	75.58	0.27	0.75	1.25	0.14
F-291	69.51	0.54	0.62	0.62	0.32
F-301-C3	73.23	0.46	0.62	1.26	0.29
M-302-C3	72.83	0.43	0.64	0.97	0.22
F-311-C4	71.81	0.41	0.69	1.68	0.21
M-312-C4	69.73	0.29	0.75	1.50	0.29
M-321	75.10	0.32	0.71	1.80	0.11
F-331	69.54	0.41	0.46	1.23	0.33
F-341	62.52	0.54	0.45	1.85	0.15

*SRI* Sleep Regularity Index, *IV* Intradaily Variability, *IS* Interdaily Stability.

What was less consistent, however, was how each night unfolded. Figure 1 illustrates this instability, with wide 95% confidence intervals for sleep duration and WASO across nearly all parents. Intraclass correlations confirm that most variability in WASO and TST came from fluctuations within the same parent across nights rather than from stable differences between parents (TST ICC = 0.27; WASO ICC = 0.12).

**Fig. 4:**
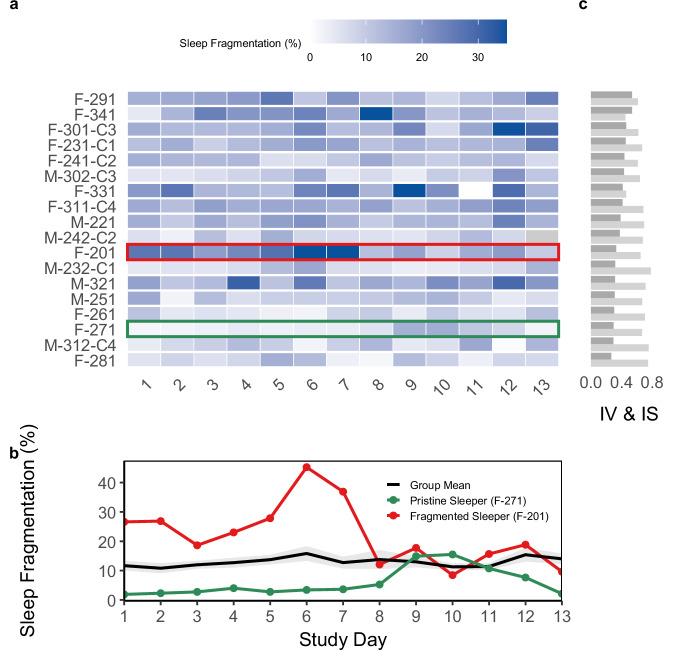
Nightly sleep fragmentation and rest-activity stability over the study period. **a** Heatmap of the fragmentation index for each parent (rows ordered by increasing Intradaily Variability, IV); darker blues indicate more fragmented sleep and lighter blues indicate greater consolidation. Red and green outlines highlight one “Extremely Fragmented Sleeper” (F-201) and one “Pristine Sleeper” (F-271), respectively. **b** Paired horizontal bars show each parent’s IV (dark gray) and Interdaily Stability, IS (light gray). **c** Line plot of mean fragmentation across all parents, overlaid with individual trajectories for F-201 (red) and F-271 (green). Peaks on days 5–6 and 12-13 reflect cohort-wide weekend-related disruptions, while individual curves illustrate markedly different vulnerability to fragmentation.

Figure 4 provides a more detailed view of these nightly fluctuations. Panel A orders parents by IV, shading each night by its fragmentation index. Parents with higher IV values generally showed more fragmented nights, while those with lower IV values seemed to exhibit more consolidated sleep. Clear individual differences were evident: some parents, such as F-241-C2, showed relatively stable fragmentation indices, while others, such as F-331, fluctuated dramatically from night to night. Figure 4c highlights the two extreme case profiles. The “Pristine Sleeper” (F-271; IV = 0.30, IS = 0.67) maintained consistently consolidated nights with fragmentation near 0–5%. By contrast, the “Extremely Fragmented Sleeper” (F-201; IV = 0.33, IS = 0.65) experienced repeated spikes reaching up to ~45%. Yet even these extremes were not fixed: by Day 9, their trajectories were alike, underscoring that fragmented sleepers can occasionally enjoy consolidated nights, and pristine sleepers are not entirely spared from disruption.

### To each their own light: interindividual differences in light exposure

**Fig. 5:**
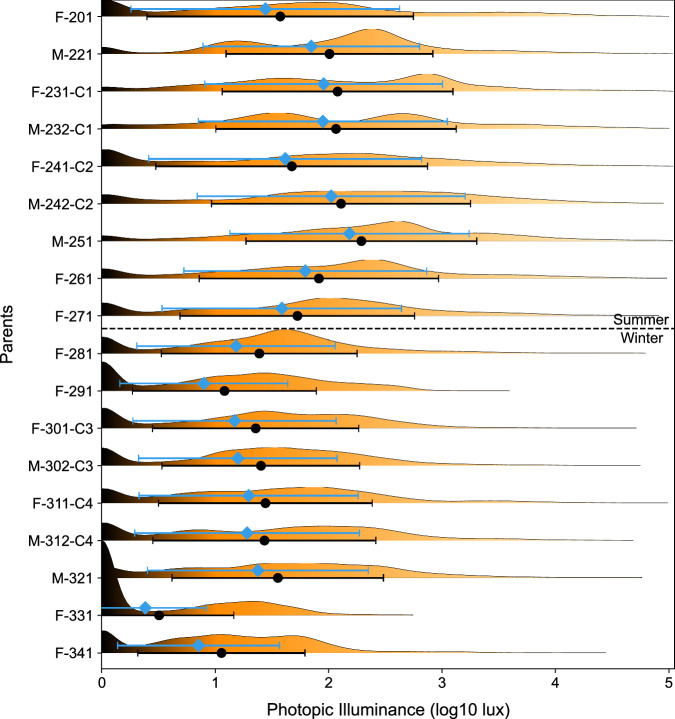
Distribution and average photopic illuminance over waking hours across parents. Black dots represent daily average photopic illuminance, and light blue dots represent daily average melanopic equivalent daylight illuminance (EDI) expressed in log lux.

Figure 5 and Table S3 indicate substantial interpersonal differences in average light exposure during waking hours. These differences between parents were confirmed by high ICCs calculated from daily light exposure metrics (mean illuminance and time-above-threshold, TAT): Melanopic EDI ICC = 0.72 log lux; Photopic Illuminance ICC = 0.69 log lux; TAT > 100 lux ICC = 0.61; TAT > 250 lux ICC = 0.64; TAT > 1000 lux ICC = 0.55; TAT > 10,000 lux ICC = 0.41 (TAT are computed on photopic illuminance). In particular, we noted differences between parents in average light exposure (over waking hours, expressed in log lux) and in average time spent above thresholds (duration expressed as percentage of waking hours >100 lux, >250 lux, >1000 lux, >10,000 lux). On average, parents spent the majority (77.65%) of their waking hours below 250 lux. While almost all parents experienced bouts of light exposure varying from 1 up to 100,000 lux, two parents (F-291, F-331) appeared to have experienced dimmer days, as indexed by a lower averaged light exposure, a smaller range, and a lack of time spent above the 1000 and 10,000 lux thresholds.

**Fig. 6:**
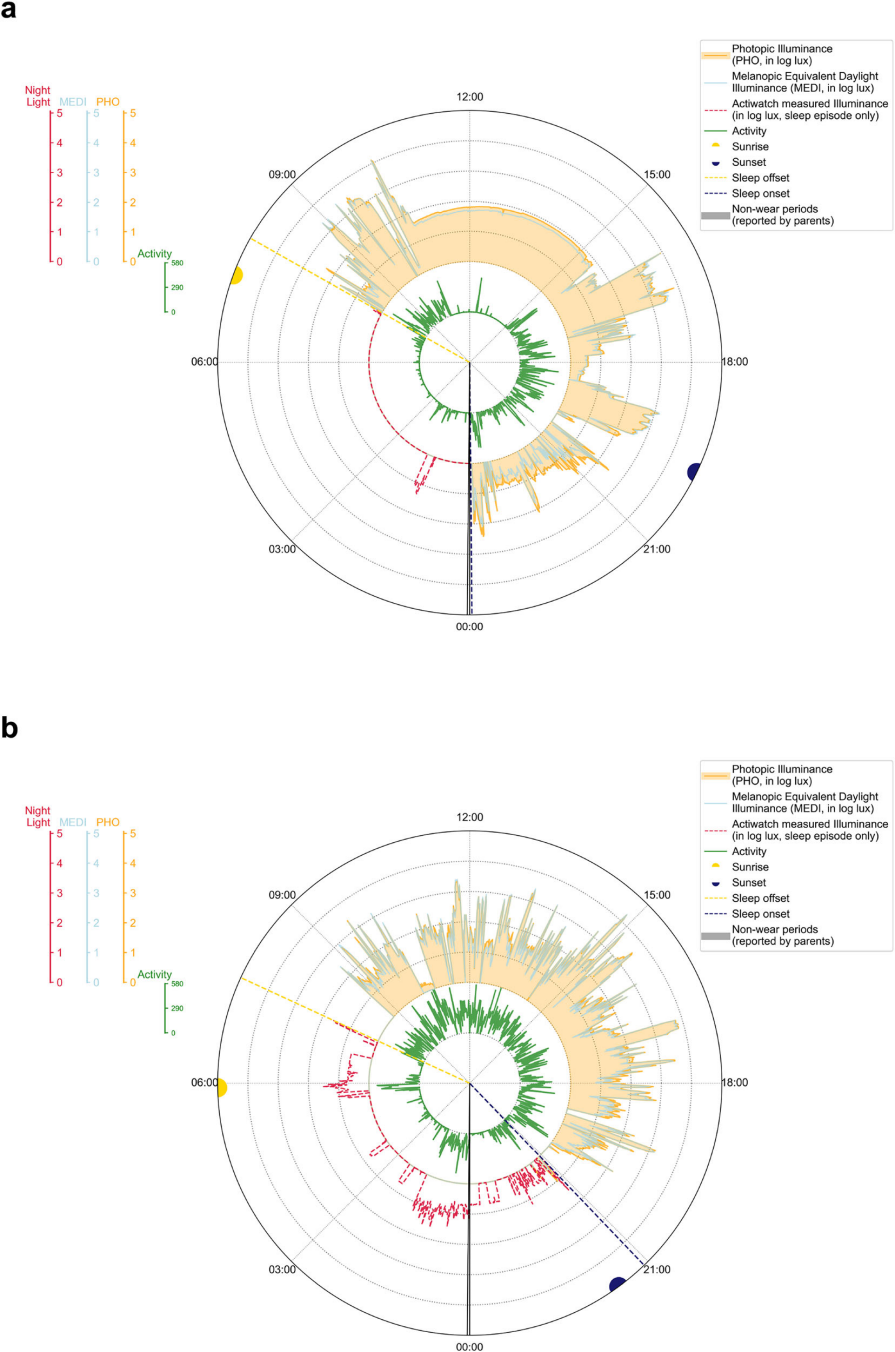
Light exposure patterns for two exemplar parents. **a** Parent F-271 (“Pristine Sleeper”) on Day 12, showing high daytime exposure with well-aligned photopic illuminance and melanopic equivalent daylight illuminance (EDI), and minimal light during sleep. **b** Parent F-201 (“Extremely Fragmented Sleeper”) on Day 7, showing multiple light exposures during the sleep period.

Average melanopic EDI was systematically lower than average photopic illuminance (see Fig. 5). However, visualizations of the light exposure throughout the days indicated a general alignment in levels of melanopic and photopic illuminance. Differences between photopic illuminance vs melanopic EDI were mostly noticeable before sunrise and/or after sunset (e.g., Fig. 6a).

Averages of light exposure during the sleeping periods (between sleep onset and sleep offset) also show differences within and between parents. Between-parent differences were indicated by small to medium ICCs calculated from light exposure metrics for the sleep episodes: Photopic Illuminance ICC = 0.34; TAT > 10 lux ICC = 0.26; TAT > 50 lux ICC = 0.18; TAT > 100 lux ICC = 0.13; TAT > 250 lux ICC = 0.05.

While the light exposure at night varied from 0 to 582 lux, most of the parents’ nights were spent in the dark with average light exposure between 1.05 and 1.82 lux (or 0.02 and 0.26 log lux). Yet, every parent experienced exposures above 10 lux, and most also above 50 lux (17/18 parents) and 100 lux (15/ 18 parents), with different durations. Table S4 informs on the average light exposure during the sleep episode per parent. Figure 6 shows 2 days of two different parents: a “Pristine Sleeper” (F-271) and an “Extremely Fragmented Sleeper” (F-201). The “Extremely Fragmented Sleeper” experienced noticeably more light during the sleep episode than the “Pristine Sleeper” (also noticeable in Fig. 3).

### Traces of seasons in daytime and nighttime light exposure

**Fig. 7:**
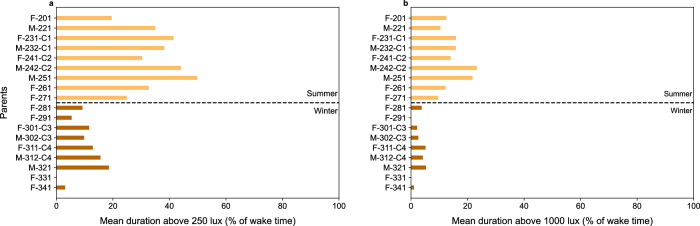
Seasonal and individual differences in light exposure timing. **a** Average time above 250 lux and **b** 1000 lux across all parents, expressed as a percentage of waking hours, separated by season.

Descriptive statistics of TATs and the timing of light exposure (see Table S3) hinted at seasonal differences between parents (Fig. 7). On average, parents monitored in winter spent less than 20% of their waking hours above 250 lux, which appeared to differ from that of parents monitored in summer (35%). This difference was also observed in time spent under lighting above 1000 lux. While the mean lighting time above 250 and 1000 lux was between 14:00 and 15:00 for parents monitored in summer, in winter the mean lighting times occurred earlier (>250 lux: mean time between 12:30 and 14:10; >1000 lux: mean time between 10:25 and 11:35). The same was observed for the timing of the last exposure at these light levels.

**Fig. 8:**
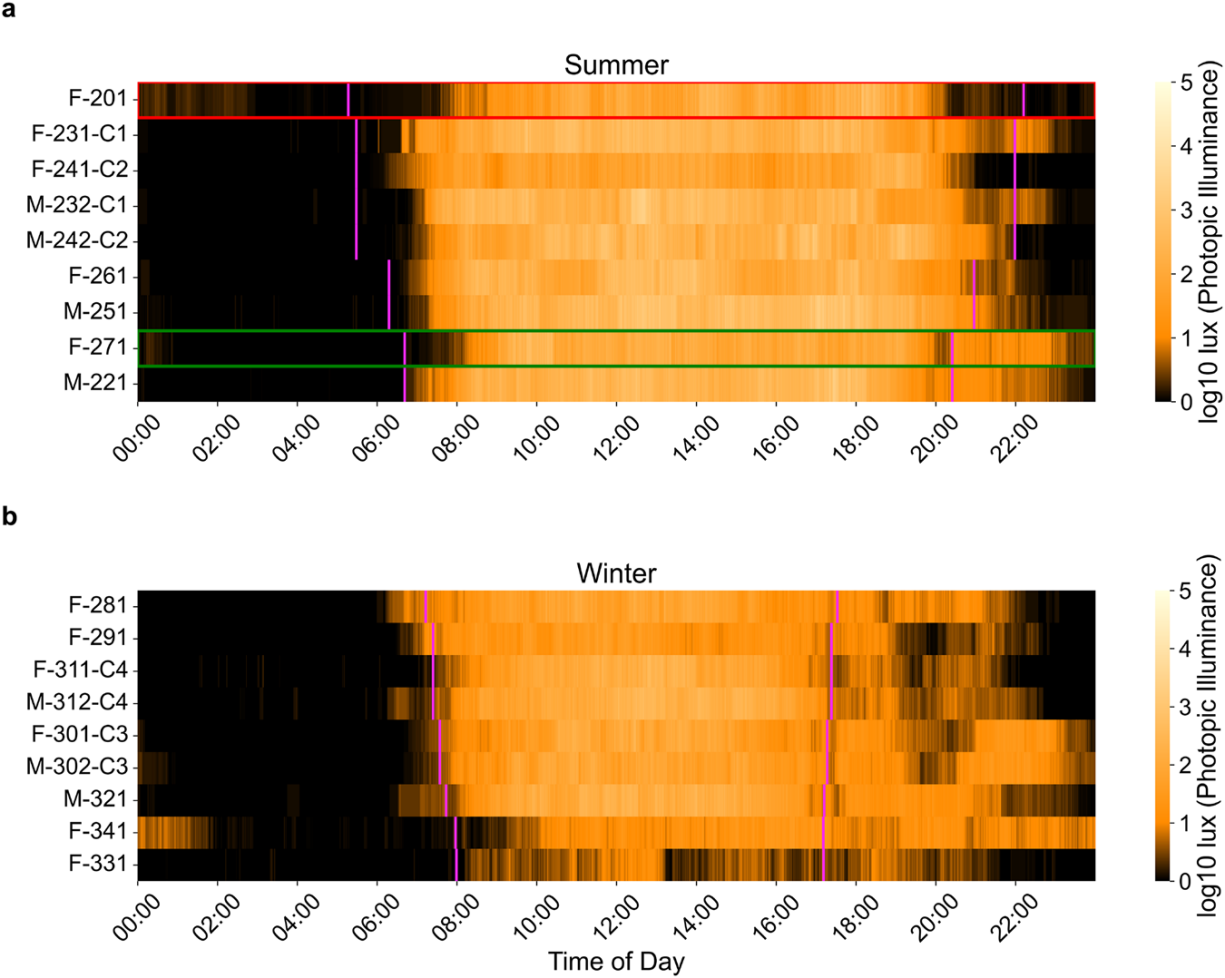
Heatmaps of an average day for each parent in summer and winter. **a** Summer days show illuminance during daytime reaching levels above 10,000 lux (10^4^ lux) and typically low levels after dusk. **b** Winter days show illuminance rarely exceeding 1000 lux (10^3^ lux), with some particularly dark days (parents F-331 and F-341). *Note:* Morning and evening pink vertical lines mark civil dawn and dusk, respectively. Green borders highlight the “Pristine Sleeper” presented previously, while red borders showcase an “Extremely Fragmented Sleeper.”

Visualizations during both daytime and nighttime also highlighted differences in light exposure between seasons (see Fig. 8 and Table S3). Parents monitored during summer days experience daytime light exposure with a wider range than parents in winter. Perhaps more notably, light exposure after dusk in summer was typically low (rarely above 10 lux), while in winter, the (artificial) light exposure reached an illuminance of 100 lux, at times blurring the line between daytime and nighttime, for example, in the case of parent M-302-C3, as Fig. 8b suggests. Daytime light exposure appeared similarly low for both the “Extremely Fragmented Sleeper” (F-201) and the “Pristine Sleeper” (F-271) (Fig. S1 in Supplementary Material).

### High variability of light exposure and temporal patterns

Despite the rhythmic nature of sleep–wake periods and photoperiods, light exposure appeared to vary substantially throughout the day and between days. Indeed, the IV measured over the study period (indexing rhythm within a day) reflects a fragmentation of parents’ light exposure, with frequent transitions. The IS of light exposure amongst parents was always below 0.33, demonstrating irregularity of light exposure on a day-to-day level. Moreover, IS and IV of light exposure appear to vary between parents (see Table 4).

**Fig. 9:**
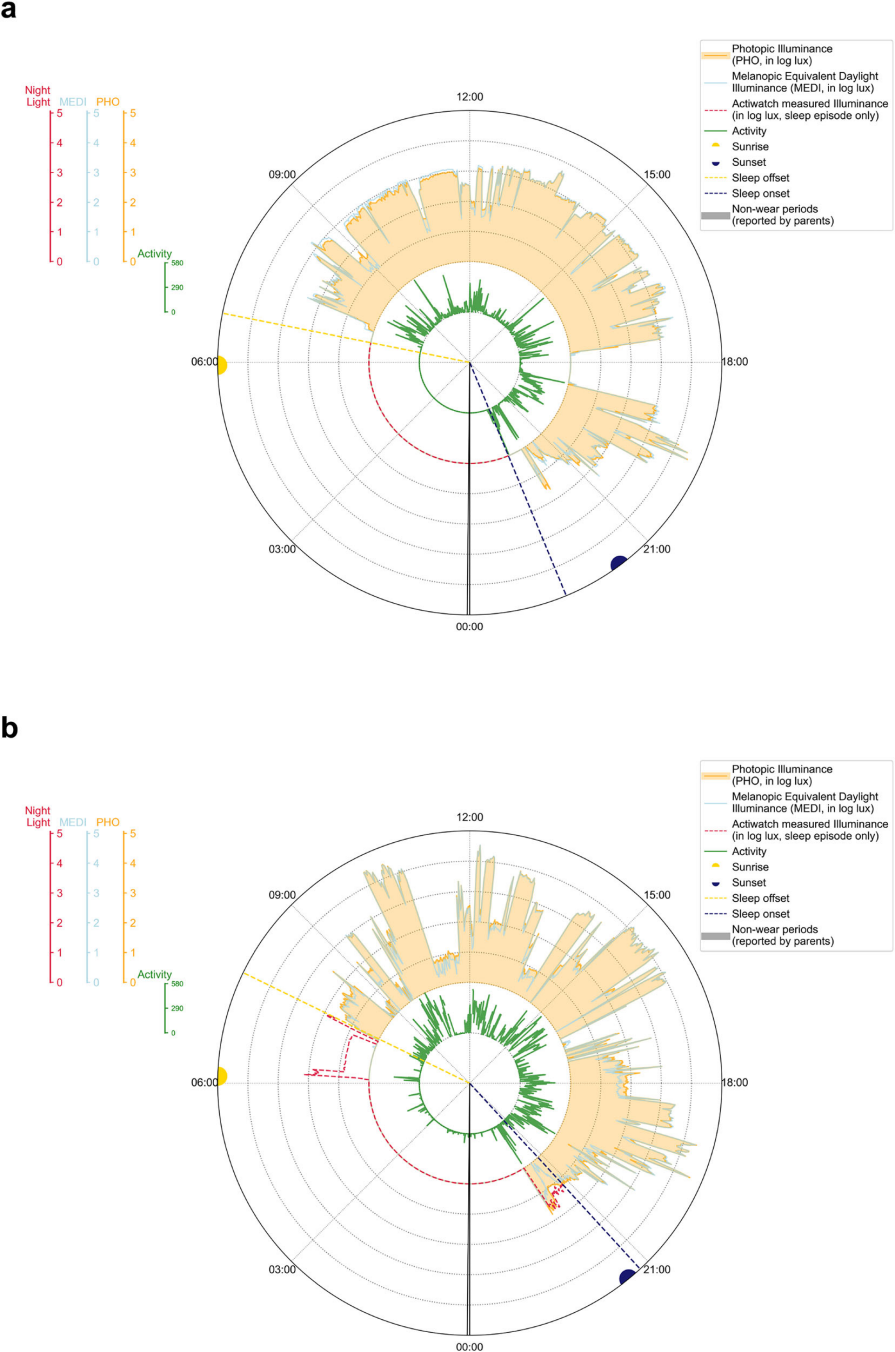
Stable vs. dynamic light exposure patterns. Comparison of a stable (**a**, Monday) vs. a dynamic day (**b**, Sunday) for parent M-242-C2.

On some days, light exposure during wake stayed rather constant (“stable days”), while on other days it varied more dynamically (“dynamic days”), as visible during in the weeks of parent M-242-C2 (Fig. 9). Some, if not most, parents (*N* = 11, F-201, M-221, F-231-C1, M-242-C2, M-251, F-261, F-281, F-301-C3, M-302-C3, F-311-C4, M-312-C4, M-321) experienced “stable days.” On the contrary, the light exposure appeared to fluctuate more frequently between dimmer and brighter levels on other days, giving the sense of “dynamic days” (see Fig. S2 in Supplementary Files for an example: an overview of “stable days” and “dynamic days” in the life of a parent). We observed this in data from multiple parents, with varying numbers of “stable” vs “dynamic” days. Additionally, for some parents, phases of “stable” vs. “dynamic” light could be distinguished within days (see Fig. S3 in Supplementary Files). Noticeably, stable and dynamic patterns of light seemed to often coincide with actimetry and, respectively, fewer or more movements (with some exceptions, as presented in Fig. S3 in Supplementary Files). Repeating patterns of stable and dynamic days in light exposure across weeks, as well as the correspondence between stable days and weekends, hinted at a link with work and work-free days (see Fig. S4 in Supplementary Files).

Patterns in light exposure also appeared when we focused on repeating time-specific peaks and dips in light exposure levels between days (see Fig. S5 in Supplementary Files as illustration). In particular, some parents experienced peaks of light exposure around noon or in the early afternoon (parents F-201, M-232-C1, F-271, F-311-C4). Perhaps more noticeably, we noted recurring dips in light exposure levels in parents’ light exposure patterns (F-261, F-241-C2, M-242-C2, F-291, F-301-C3, M-302-C3, F-311-C4, M-312-C4, F-341) often occurring in the afternoon or in the evening.

### How the baby can shape parental sleep and light exposure

Parental sleep and light patterns seemed to vary slightly depending on the baby’s sleeping location, age, and breastfeeding status, although differences in this sample were small (see Fig. 10 in Supplementary Files). While total sleep duration appears similar for parents who room-shared and those whose baby slept in a separate room, room-sharing parents tended to spend somewhat more time awake during the night. Parents of younger infants (<6 months) generally slept longer than those with older infants, but this was accompanied by more frequent and prolonged nocturnal awakenings. A similar pattern could be observed for breastfeeding parents, who slept slightly longer but also spent more time awake after sleep onset compared to non-breastfeeding parents.

**Fig. 10:**
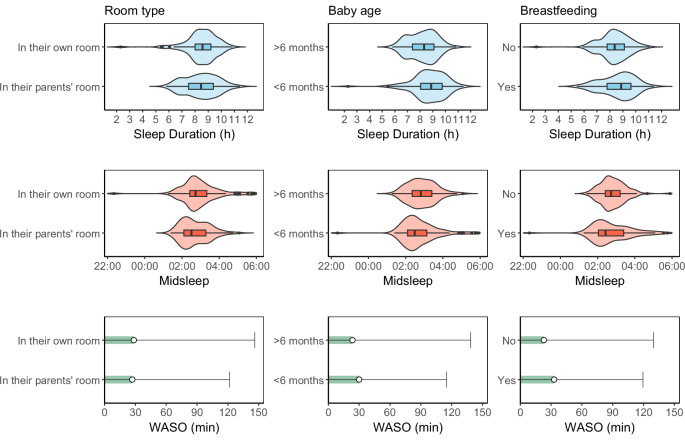
Parental sleep duration, midsleep timing, and wake after sleep onset (WASO) in relation to the baby’s sleeping location, age, and breastfeeding status. Violin plots show the distribution and median values for sleep duration (top row, blue) and midsleep timing (middle row, red). Error bar plots (bottom row, green) display means and 95% confidence interval for WASO. Results indicate modest differences in total sleep duration by sleeping arrangement, clearer effects of infant age on midsleep timing and WASO, and increased nocturnal wakefulness among breastfeeding parents.

These patterns were also visible in light exposure (see Fig. 11 in Supplementary Files). During sleep episodes, the time spent by parents in a light environment above 10 lux seemed, on average, slightly longer when the baby slept in their own room, compared to in the parental room, consistent with the need to get up during the night. Yet, individual cases highlighted that group averages could be misleading. Parents F-261 and F-281 spent on average less than 3% their sleeping periods above 10 lux, while parent M-321 spent on average 10% of his sleeping period above 10 lux. All three parents (F-261, F-281, M-321) slept with their babies in their room. By comparison, parent F-201—whose baby slept in the parental bed—had the highest light exposure during sleep with an average of 10.73% of her sleeping periods in a light environment above 10 lux.

**Fig. 11:**
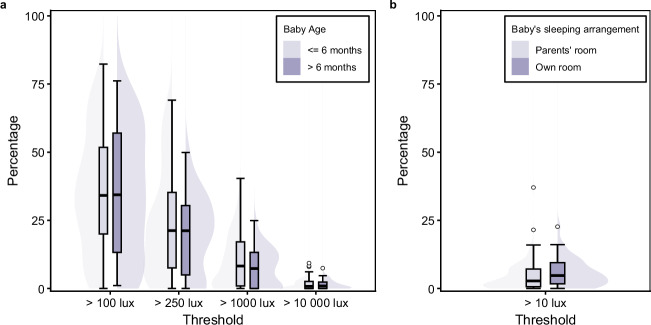
Light exposure of parents in relation to their baby’s age and sleeping location. **a** Differences in average time above threshold between parents of a 3–6-month-old baby (light violet) and 6–12-month-old baby (dark violet); time above threshold is expressed as a percentage of time awake. **b** Average time above 10 lux during sleep episode of parents with their infant sleeping in parents' room (light violet) and in their own room (dark violet). Time above threshold is expressed as a percentage of time asleep.

Average daytime light exposure seemed not to or only subtly differ between parents of younger (3–6 months) versus older (6–12 months) infants (Fig. 11a) as the average times above threshold at 100, 250, 1000, and 10,000 lux did not show a clear difference between these groups (Fig. 11a).

### Gender differences in sleep and light exposure

**Fig. 12:**
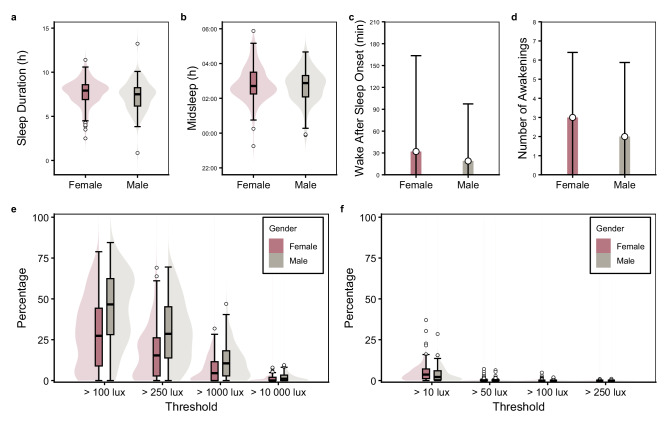
Sleep and light exposure across gender. The top row panels show, for each gender, the median, 95% confidence interval, distribution, and outliers of **a** Sleep Duration, **b** midsleep, **c** wake after sleep onset (WASO), and **d** number of Awakenings, based on actigraphy-recorded sleep episodes. Bottom row panels show gender differences in average time spent above illuminance thresholds during **e** wake and **f** sleep episodes.

At the group level, mothers’ sleep seemed slightly more burdened by nighttime disruptions than fathers’ sleep (Fig. 12, Table S5). Visual inspection suggests that mothers experienced, on average, more WASO than fathers, and their nights contained a median of three awakenings compared to two in fathers. Bed- and wake-times were nearly identical, and both groups spent comparable time in bed and time asleep (TST). Subjective ratings of quality and restedness were also rather similar. Looking within couples, this pattern was also visible (see Fig. S6 in the Supplementary Files). In every mixed-gender dyad (Couples 1, 2, 3, and 4), fathers’ sleep was more consolidated, while mothers endured more frequent or prolonged disruptions. Interestingly, the gender differences in sleep consolidation as measured using actigraphy did not carry over into parents’ own diary reports of subjectively experienced awakenings (see Fig. S7). Both fathers and mothers recall nearly the same number and duration of awakenings, suggesting a potential difference in the perception of sleep between genders.

**Fig. 13:**
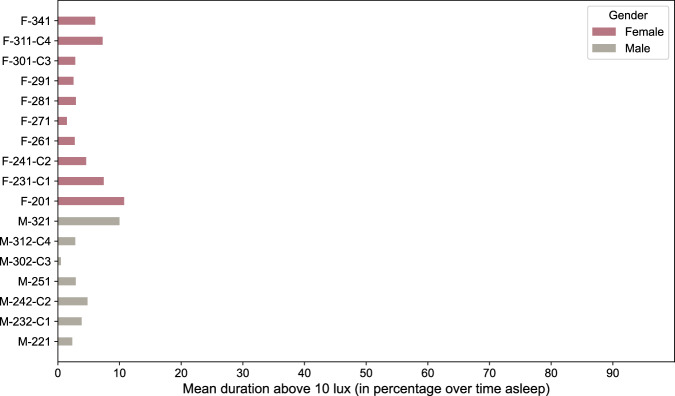
Individual average time above 10 lux during sleep episodes. Mothers are represented in deep red and fathers in gray.

**Fig. 14:**
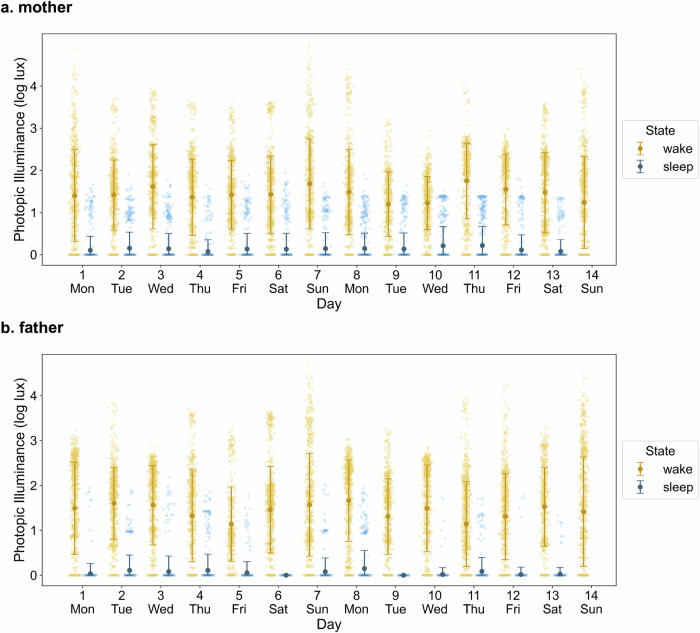
Two-week light-exposure patterns in a mother and father. Light exposure and distribution of light exposure over 2 weeks of a mother (**a**) and a father (**b**).

Across the sample, all parents encountered at least some light above 10 lux at night (Figs. 12f and 13), but mothers did so slightly more often and for longer than fathers. Indeed, when observing both parents of the same infant (couples 1, 3, and 4), we noted more occurrences of light exposure during the sleep episode in mothers. In particular, in couple 4, the mother (F-311-C4) experienced light exposure above 10 lux every night, a striking opposite to the father (M-312-C4), who experienced mostly dark nights (Fig. 14). In couple 2, we observed the opposite tendency, whereby the father experienced a few light exposure episodes during sleep, while none were found in the mother’s data. However, in this couple dynamic, the light exposures of the father during sleep episodes were mostly close to sleep onset or sleep offset.

Daily rhythms of light and rest revealed both contrasts and alignments between mothers and fathers. One notable difference was daytime napping. While mothers did not nap more frequently than fathers, their naps were generally longer. This pattern was also reflected within couples: mothers typically napped longer than fathers, with one exception, and one father did not nap during the study period.

Inspection of light exposure showed another type of gender difference. Fathers generally spent more time in brighter environments, with longer exposures above 100, 250, and 1000 lux during waking hours (Fig. 12e). At the highest intensities, above 10,000 lux, however, mothers and fathers looked alike.

**Fig. 15:**
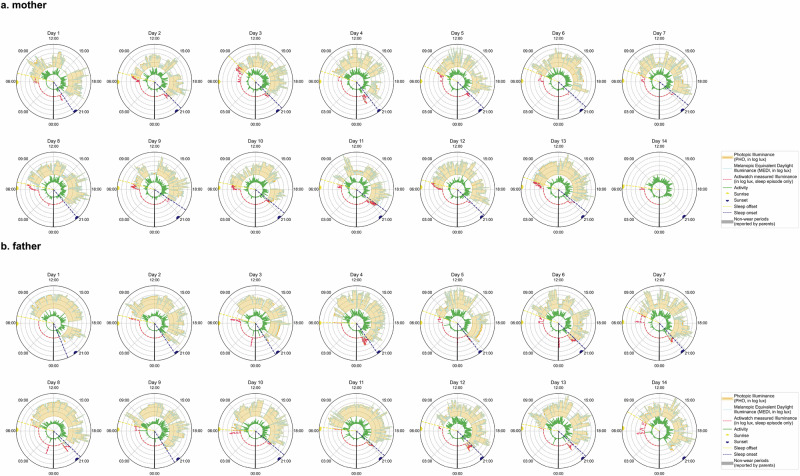
Two-week light-exposure patterns in couple F-241-C2/M-242-C2. Light exposure over 2 weeks in the F-241-C2/M-242-C2 couple: a mother (**a**) and a father (**b**).

**Fig. 16:**
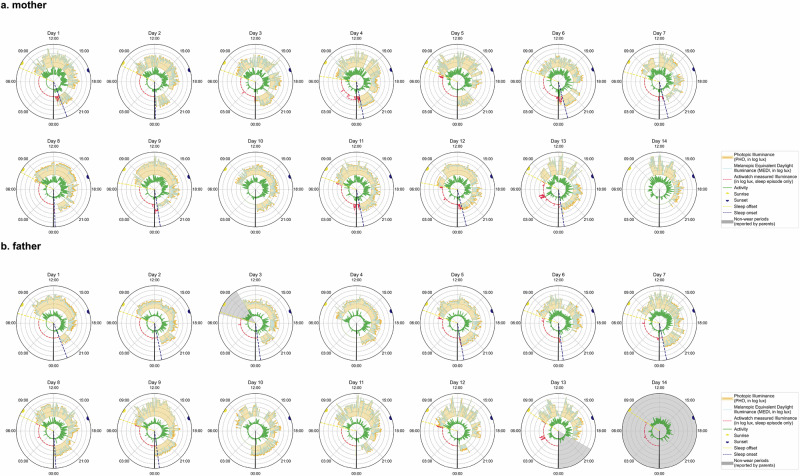
Two-week light-exposure patterns in couple F-301-C3/M-302-C3. Light exposure over 2 weeks in the F-301-C3/M-302-C3 couple: a mother (**a**) and a father (**b**).

At the same time, couple-level patterns reveal how shared routines can synchronize parents’ rhythms. In couple 2, for example, both parents showed dips in light around 09:00 and 15:00, sometimes on the same days and sometimes only in one parent (e.g., the father on day 13, the mother on day 11; Fig. 15). Similarly, couple 3 displayed a recurring evening dip between 20:00 and 21:00, noticeable almost every night in both parents, though occasionally visible in only one (Fig. 16). These repeated dips pointed to moments of alignment.

## Discussion

Night-to-night fluctuations in sleep within and between parents of young infants, as well as variations of the light environments in which they unfold, have been largely undocumented. By combining actigraphy, diaries, and wearable light sensors across 2 weeks in both mothers and fathers of infants aged 3–9 months, our study provides a detailed ecological portrait of sleep and light exposure patterns in early parenthood. Our deliberately descriptive approach allows us to capture the volatility of nightly sleep and daytime rest, and overlaps and discrepancies between objective and subjective reports of sleep disturbances. Similarly, a focused description of light exposure enables us to note the diverse, often irregular, light ecologies experienced by parents, extending beyond seasonal differences to complex light patterns with both stable and dynamic days. Rather than averaging out variability, we sought to highlight it, and in doing so provide an empirical foundation for circadian-aware inference and intervention.

### Sleep of parents with young infants: disrupted and highly variable

Parental sleep in the first year of their baby’s life is consistently fragmented and less consolidated than before birth^15,19,48,49^. Our descriptive findings suggest that parental sleep might not be *too short* as much as *disrupted*. Although parents often obtained total sleep durations that met or exceeded recommended guidelines (average TST ≈ 8.5 h;^50^), these totals masked considerable fragmentation: on average, 2–3 awakenings and ~40 min of WASO per night. While this frequency of awakenings is comparable to parent-reported infant night-waking frequencies in infants aged 3–9 months^51^, it substantially exceeds normative sleep continuity observed in healthy adults of a similar age (<20 min WASO;^47^). In practice, this meant that several parents achieved little more than four consecutive hours of uninterrupted sleep, a threshold suggested as the minimum required for restorative recovery across NREM-REM cycles^52^. These results echo prior findings that in parents of infants under 1 year, the main disturbance is not TST but disrupted continuity, with frequent awakenings and prolonged WASO, suggesting that parents compensate for expected nighttime wakefulness by spending more time in bed (i.e., going to bed earlier)^4,15,19,48,53,54^. This pattern has been described as *negotiated sleep*—an adjusted sleep behavior adopted to face the demands of infant care, partner coordination, and household routines, rather than determined by individual physiological need alone^4,55,56^.

Although general sleep disturbances in parents of young infants are well established, their *instability* has received far less attention. Our descriptive mapping illustrates the profound heterogeneity of parental sleep: some parents maintained nearly pristine, consolidated nights, while others endured persistent fragmentation, and many oscillated between those extremes across successive nights. This mirrors evidence of substantial between-parent differences in sleep continuity during the postpartum period^19,48,53^, but also growing recognition of within-parent, night-to-night variability as an important dimension. Kalogeropoulos et al.^57^ quantified both sources of variability in parents of 6-month-old infants. They reported high *within-parent* coefficients of variation (0.25–1.32) for the longest consecutive sleep bout and number of awakenings across ten nights. Additionally, they identified a *between-parent* subgroup—about one quarter of parents—who achieved six or more hours of uninterrupted sleep on fewer than three nights, closely resembling what we see in the illustrative cases of “extremely fragmented sleepers”^57^. Consistent with these findings, our data revealed comparable instability: variance in TST (ICC = 0.27) and WASO (ICC = 0.12) arose predominantly within parents across nights, accompanied by high Intradaily Variability (IV = 0.36 ± 0.10). In conclusion, these results suggest that for many parents, postpartum sleep is characterized less by chronic restriction than by marked instability and irregular consolidation across successive nights.

High fragmentation and variability of parental sleep may carry important consequences for health, mood, and caregiving. Fragmented sleep has been repeatedly associated with elevated depressive symptoms, poorer mood regulation, fatigue, and impaired cognitive performance in postpartum parents, with indices of sleep continuity such as WASO and the number of awakenings emerging as stronger predictors of depressive symptoms than total sleep duration^10,21–23^. Sleep fragmentation has also been linked to lower maternal sensitivity and responsiveness during mother-infant interactions, suggesting that disrupted restorative sleep may compromise caregiving quality^10,58,59^. Likewise, night-to-night variability in sleep duration and timing has emerged as a distinct marker of health risk: greater instability predicts poorer mood, greater daytime fatigue, and diminished emotional functioning in postpartum and general populations^23^. Collectively, this body of evidence indicates that both fragmentation and variability are not merely descriptive features but potential mechanisms linking disrupted parental sleep to compromised well-being and caregiving quality in the postpartum period, which could be investigated in future studies.

### Daytime rest and activity rhythms

Beyond the nocturnal sleep period, extended periods of daytime inactivity were nearly ubiquitous in this cohort. Actigraphy revealed that nearly all parents engaged in daytime rest bouts, often exceeding 5 h in total per day. Subjectively, 80% of parents reported that they napped during the day, with a mean duration of 49.9 min (±54.6). Descriptive inspection showed, once again, a wide heterogeneity: some parents relied on multiple short rest bouts scattered across the day, others on longer consolidated periods of rest, and many alternated between strategies. Importantly, these patterns seemed not to be systematically linked to nights of more fragmented sleep or to parents labeled as overall “extremely fragmented” versus “pristine” sleepers.

Daytime rest in early parenthood thus appears to function as a flexible, context-driven adaptation rather than a direct response to nocturnal disruption. The literature similarly indicates that naps are common among postpartum parents but are only inconsistently restorative. While some studies report temporary reductions in fatigue following daytime sleep^60^, others show that naps do not reliably improve SSQ or mood and may even coincide with poorer nocturnal efficiency or persistent tiredness^61,62^. Actigraphic evidence further reveals that, although rest bouts during the day are widespread, they only partially offset sleep fragmentation and do not restore baseline alertness^48^. These mixed outcomes likely reflect the opportunistic nature of parental rest—brief, unplanned, and often dictated by infant sleep patterns rather than deliberate recuperation.

Adding to this complexity, daytime inactivity is frequently misclassified or underreported depending on the measurement approach. In our data, many extended low-activity periods were not identified as naps in diaries, consistent with previous work showing systematic differences in subjective and objective sleep in postpartum samples^11,48^. In general adult populations, similar discrepancies arise because actigraphy tends to overestimate sleep during quiet wakefulness or sedentary activity^63–65^. Even more advanced sensing modalities face similar challenges: while polysomnography (PSG) remains the gold standard for sleep staging, its application in naturalistic daytime settings is rarely feasible, and alternative approaches—such as portable EEG or multimodal wearables—have not yet been systematically validated for detecting daytime naps^66,67^. Together, these findings highlight the methodological and conceptual ambiguity of “daytime rest” in early parenthood: actigraphic inactivity may represent a blend of genuine recuperation, passive caregiving (such as holding a sleeping infant), and non-sleep quiet time.

### The importance of adequate light

Sleep disruptions occurred in the context of varying light exposure profiles (varying both between parents and within parents between days), a fact that, in earlier research among parents of young infants, has often been overlooked. Over the week, days were never the same, due to environmental conditions (weather, for example) but also due to social (and possibly parental) constraints. Indeed, we noted important individual, day-to-day, and within-day variability in light exposure. Intraclass correlations for daily light metrics were high (ICC ≈ 0.61–0.69), reflecting stable between-person differences. Yet, Interdaily Stability was rather low (IS < 0.33) and Intradaily Variability high (IV between 0.97 and 1.85), revealing that individual parents’ day-to-day light patterns differed markedly.

According to our results, most parents spent the majority of their waking hours in a dim light environment—most probably indoors—under 100 or 250 lux (photopic illuminance), with only a few occurrences of light exposure above 1000 lux. Light exposure during the sleep episode was a common occurrence in our sample of parents, with illuminance ranging from 0 to 582 photopic lux and averages of 0.49–10.73% of sleep time spent above 10 lux. Our descriptive results on wake-time light exposure align with prior averages recorded by Tsai et al. and Lee et al. among mothers (respectively reporting 71.13% of wake time spent under 50 lux and averages of 73 lux)^16,44^. Light exposure during sleeping periods, however, appears marginally brighter in our sample than in prior studies^44^. Contrary to earlier findings^16,44^, almost all parents included in our study (17/18) experienced bright light exposure above 1000 and 10,000 lux, with a difference in time above these thresholds between parents monitored in the summer and in the winter. This observation hints at an impact of seasonality on light ecologies: a question already raised by Goyal et al.^46^, which merits further investigation.

Comparing the light exposure of parents with young infants in our study to current recommendations highlights the possible adverse effects on their sleep and overall well-being. According to Brown et al., light exposure during daytime should exceed 250 lux (melanopic EDI) at eye level to support homeostasis and maintain a healthy circadian rhythm^37,38,68^. In the evening, light exposure should not go beyond 10 lux (melanopic EDI), and during sleep, it’s recommended to stay below 1 lux (melanopic EDI) as research has shown that even brief nocturnal bursts above 10 lux can suppress melatonin secretion, which in turn has the potential to prolong nightly awakenings and shift circadian rhythms^69,70^. In comparison, the light exposures during waking hours and sleep episodes experienced by the parents monitored in our study deviated from the recommendations.

Nighttime light exposure may be a relevant concern in our sample of parents. Parents (perhaps mothers in particular) frequently experienced brief nocturnal intrusions of light >10 lux during infant care, which seemed to often align with longer WASO. As parents of young infants experience more and longer awakenings after sleep onset, most probably due to infant care, they may be more vulnerable to circadian disruption as a result. Disturbed circadian rhythms are increasingly recognized in new parents, with studies showing reduced Interdaily Stability and elevated variability in actigraphy-derived rhythms during the first postpartum months^23,57,59^. Such instability is not only associated with disrupted sleep but has also been linked to greater fatigue, reduced alertness, increased anxiety, and mood disturbance in both general and perinatal populations^23,71,72^. Yet, while we can advise parents to keep a light exposure below 10 lux during night feeding or infant care, awakenings (and resulting light exposure) are bound to occur and can disrupt sleep and sleep–wake rhythms.

Another notable observation was the relatively dim light environments experienced by parents during waking hours. Insufficient daytime light exposure is known to blunt circadian amplitude, undermining consolidated rest, even in undisturbed sleep conditions^73–75^. In the face of sleep disruption experienced by parents, the amount of daytime light exposure (both in duration and intensity) appears even less prone to correct for the impacts of sleep, nightly awakenings, or nightly light exposure on their circadian rhythm. The importance of (bright) light during daytime also relies on its mood-enhancing and depression preventing action^76–78^. In our sample, parents were exposed to only a few and brief illuminances above 10,000 lux: a level commonly used in bright light therapy^79^. The instability we observed in both sleep and light ecologies of parents with young infants likely carries consequences for parental well-being that extend beyond the night. Parents in our sample presented high scores on the Edinburgh Postnatal Depression Scale (EPDS), hinting at depressive symptoms, perhaps in link to parental dim light ecologies: a question that merits further investigation.

To better understand postpartum sleep and circadian disruptions and their numerous effects on parents with young infants, future research should examine the reciprocal disruption occurring between sleep and light, which can only be observed when both factors are monitored together over time. At present, we advise parents to increase daytime outings, enabling, in turn, exposure to higher light intensity (even in winter)than common indoor environments provide. Such behavioral change also benefits infants, for whom light is equally essential for circadian entrainment^80^.

### Going beyond light exposure thresholds and averages

Additionally, our descriptive visualizations revealed diverse ecological patterns in light exposure. Some parents showed stable wake-time profiles, whereas others had more dynamic profiles with frequent transitions and higher illuminances. These patterns sometimes differed between relatively stable “weekdays and more dynamic” weekends, or even occurred within the same day. Recurring features included midday peaks above 1000 lux, possibly reflecting lunch outings, morning and late-afternoon peaks consistent with commuting, and evening dips near infant bedtime. Such regularities and irregularities are not captured by aggregate metrics such as “time above 250 lux,” but become evident through descriptive and visual profiling of time-series data. Recent work has begun to examine light regularity as a potential new metric^81,82^. Although the light regularity index is new and not yet validated, it represents a step toward better characterizing light ecologies.

These observations highlight the shortcomings of current metrics, as no adequate measure can reliably and completely capture the richness of these patterns. Indeed, thresholds tell only part of the story. Two parents may both accumulate ~3 h above 250 lux, but one receives this in a continuous morning block, and the other in irregular bursts later in the day. Such profiles differ profoundly in circadian impact^83^, yet scalar metrics do not distinguish them. Time above thresholds (TAT) only references to durations, while mean lighting time above threshold (MLit, as well as first and last timing above threshold) refers to time-of-day and benchmark thresholds^84^. Currently, no metrics are able to encompass time-of-day, illuminance, and duration in one. However, time of day as well as duration of light exposure are essential factors of bright light and its effects^84^. This was emphasized by studies on light therapies (or on the impact of bright light exposure)^77^, resulting in the recommendation of bright light exposure (above 500 or 10,000 lux) in the morning^37^.

Together, these cases highlight the heterogeneity of parental light environments, not only in amount but also in timing, structure, and regularity. While photopic illuminance and melanopic EDI can inform light ecologies, the spectral composition of the light may also play a role. In addition to exploring light ecologies in detail over time, future studies should explore the spectral diets^85,86^ of parents with young infants.

### Infant factors, gender roles, and couple dynamics

Sleep and light exposure patterns also varied according to infant characteristics and caregiving roles. Parents of younger infants and breastfeeding mothers slept slightly longer (TST), but experienced more nocturnal awakenings, consistent with prior findings that infant age and feeding method shape both maternal and paternal sleep continuity^49,87^. Parents who shared rooms with their infants showed lower sleep continuity (e.g., higher WASO and shorter longest sleep period) and later sleep timing in several cohorts, echoing evidence that infant sleep location influences parental sleep continuity and timing independent of the infant’s age^49,88,89^. These differences were mirrored in light exposures: for example, parents who needed to get up for infant care in a separate room accumulated more nocturnal light, highlighting an interplay between caregiving arrangements and circadian disruption.

Gender roles also seemed to shape these sleep-light ecologies. The results of our descriptive analyses revealed that mothers might wake more often and accumulate more nocturnal light exposure than fathers. This aligns with field studies showing mothers experience more fragmented nighttime sleep and closer coupling to infant awakenings than fathers, whose sleep is typically more consolidated (though sometimes shorter in duration)^4,19,90^. In contrast to mothers, fathers tended to overestimate their WASO more in sleep diaries (compared to actigraphy-derived estimation), suggesting asymmetries not only in burden but also in perception of sleep disruption. Within-couple comparisons in our data showed synchrony in shared bed- and wake-times but also divergence in fragmentation and nocturnal light—underscoring the value of treating the household as the unit of analysis rather than focusing on a single parent.

### Limitations

Several limitations of this study should be acknowledged. First, the sample was modest in size and relatively homogeneous in terms of socioeconomic and cultural background, which limits generalizability. In addition, the sample size and descriptive nature of the study limited our ability to formally examine or control for potential confounding factors that may contribute to the substantial variability observed in sleep and light exposure patterns, such as caregiving arrangements, work schedules, infant characteristics, or environmental circumstances. Second, the 2-week recording window offers only a snapshot of the postpartum period and may not reflect longer-term adaptations. Third, wrist-worn and chest-level devices capture illuminance at that specific location, and only allow for an estimation of light exposure at eye level. As such, our monitoring method might underestimate or mischaracterize light exposure due to occlusion, spectral sensitivity, or placement. Similarly, actigraphy infers sleep from movement and can misclassify quiet wakefulness, caregiving immobility, or co-sleeping as sleep. In addition, algorithmic choices such as the activity-count threshold, minimum sleep-bout duration, permitted wake gaps within sleep bouts, and diary-constrained sleep windows directly influence which periods are classified as sleep. Unfortunately, our sample of mothers and fathers included only four couples, which provided only hints at dyadic interactions. These constraints should be kept in mind when interpreting our descriptive findings and underscore that the results provide an ecological portrait rather than precise estimates of associations or causal mechanisms.

### Moving from descriptives to inferences: future studies

Our findings underline why descriptive analyses must precede inference in this field. By mapping variability in sleep, light, and rest-activity rhythms, we have shown that parental ecologies are highly diverse, often unstable, and shaped by seasons, infant care, gender roles, and household routines. These descriptive portraits are not an endpoint but the foundation for future work. They highlight heterogeneity that averages obscure, they ground ecological validity by showing where self-reports and objective measures diverge, and they generate hypotheses for statistical testing.

The next step in investigating the relationship between light exposure and sleep among parents of young infants involves characterizing the temporal dynamics of their bidirectional effects^18^: for example, to what extent does low daytime illuminance influence subsequent nighttime sleep duration and fragmentation, and how, in turn, does sleep quality shape light exposure behaviors the next day? Additionally, future studies should link daily light ecologies and nocturnal light intrusions to sleep fragmentation and daytime outcomes such as fatigue, sleepiness, and mood, taking care to use multilevel and/or time-series models that capture between-day^18^, within-person^23,91^, and between-person variability. Moving beyond descriptive profiles to phase-referenced analyses is essential because it allows us to investigate whether light exposures occur at sensitive circadian windows that amplify their impact. Such work can furthermore clarify mechanisms through which instability in sleep-light patterns contributes to impaired functioning and elevated mood disturbances in the postpartum period^23,72^.

Another important direction within this field is circadian-aware inference^92,93^. Anchoring observations to circadian phase markers will enable a more mechanistic understanding of how parental ecologies disrupt circadian timing. Intervention studies can then assess whether approaches such as morning bright light, evening dimming, or minimizing nocturnal exposure during infant care improve both sleep consolidation and daytime functioning. Given our results, such interventions may need to be tailored to the individual, considering caregiving role, infant age, season, and other contextual factors. In this way, descriptive studies such as ours lay the groundwork for translational advances in parental sleep and circadian health.

## Methods

### Study design

This paper reports descriptive findings on light exposure and sleep patterns of parents with infants between 3 and 9 months old, as collected in the SUNSHINE project. The primary aim of this study was not to test predefined hypotheses or estimate population-level effects, but rather to characterize real-world sleep and light ecologies in a naturalistic postpartum context. Given the exploratory nature of the project and the relatively small and heterogeneous sample, the study was designed to provide detailed descriptive profiling and to generate hypotheses for future larger-scale investigations.

The study followed parents for 14 consecutive days from Monday to Sunday using an observational design. Sleep patterns were evaluated using daily sleep diaries and actigraphy (MotionWatch8, CamNtech). Light exposure was assessed using wearable light exposure sensors worn on collars (ActLumus, Condor Instruments) and a light sensor inside the wrist-worn Actiwatch (considered for light exposure during sleep episodes). Data on infant sleep behavior, parental sleep quality, depressive symptoms, daytime sleepiness, chronotype, and perceived stress were gathered at an intake appointment, a maximum of 2 weeks before the observation period started. Additional measures were collected as part of the broader study protocol, including repeated ecological momentary assessments of positive and negative affect, daytime sleepiness, energy levels, concentration, and tiredness throughout the day, as well as continuous skin temperature monitoring using iButtons to estimate circadian rhythmicity. These outcomes will be reported in separate publications.

### Participants

A total of 19 parents (11 mothers and 8 fathers, including 4 couples) with infants aged 3 to 9 months were recruited through online advertisements, Dutch university networks, and flyers distributed at daycares in the Eindhoven region. Participation as a couple was encouraged. Eligibility criteria excluded individuals diagnosed with narcolepsy, psychotic disorders, or bipolar disorders, as well as those who were not regular smartphone users or fluent in English. Due to very low protocol compliance (<10% of the expected data collected), one parent was excluded from the analyses, resulting in an analytical sample of 18 parents. Participants received 85 euros for participation, with an additional 15 euros charity bonus awarded when at least 75% of assessments were completed during the second study week.

Throughout the manuscript, parents are identified using a code indicating Gender-Participant Number-Couple Identification (e.g., M-232-C1 refers to male participant 232, who was monitored together with partner F-231-C1). For parents whose partner did not participate, only gender and participant number are reported.

### Measures and procedure

#### Intake

During the intake appointment, parents received detailed information about the study protocol and comprehensive instructions for wearing the sensors—including proper positioning, attachment, and troubleshooting. In the same session, they completed self-report questionnaires administered via LimeSurvey to assess demographics, infant sleep patterns and behaviors, parental sleep quality, chronotype, depressive symptoms, daytime sleepiness, and perceived stress, using the Infant Sleep - Brief Infant Sleep Questionnaire-Revised (ref. ^94^), PSQI (ref. ^95^), ultra-short Munich Chronotype Questionnaire (*μ*MCTQ; ref. ^96^), EPDS (ref. ^97^), Epworth Sleepiness Scale (ESS; ref. ^98^), and Parental Stress Scale (PSS; ref. ^99^). Detailed descriptions of all questionnaires are provided in Supplementary Materials S1.

#### Sleep assessment

Parental sleep during the 2-week observation was assessed using both subjective and objective methods. Parents completed a daily sleep diary and wore a wrist-mounted actigraph (MotionWatch 8, CamNTech) continuously. Each morning at 07:00, the *Consensus Sleep Diary—Morning version* (CSD-M;^100^) was delivered via the M-Path mobile application (KU Leuven) and remained available until the end of the day. The diary captured self-reported bedtime, try to sleep time, sleep onset latency (SOL), wake-up time, time out of bed, number and total duration of nocturnal awakenings (WASO), and SSQ. Additionally, the MotionWatch 8 recorded tri-axial accelerometer data—aggregated into activity counts—in 30-s epochs to provide objective measures of rest-activity patterns.

#### Light exposure measurements

Light exposure was monitored during the 2-week observation period using two complementary devices. Daytime exposure was assessed with personal light sensors (ActLumus, Condor Instruments) worn as chest-level pendants. Parents were instructed to wear the sensors during all waking hours, to place them on their nightstand with the light-receiving surface facing upward during sleep, and to reattach them whenever leaving the bed (e.g., to care for their infant). The ActLumus sampled at 1/60 Hz across ten spectral channels, which were combined to derive estimates of photopic illuminance and melanopic equivalent daylight illuminance (EDI, both in lux). Light exposure during the sleep episode (between sleep onset and sleep offset) was also assessed using the integrated sensor of the MotionWatch 8 (CamNtech Ltd.), which was worn continuously on the wrist. This device recorded photopic illuminance in 30-s epochs. No device-specific calibration was performed prior to the usage of the ActLumus or MotionWatch 8.

### Data processing

All processing and visualizations were performed in Python (v 3.12.7) and in R (v.4.5.0).

#### Sleep diaries

Overall, 6.4% diaries were missing or incomplete; these days were excluded from all analyses rather than imputed. Sleep Onset was calculated by adding the SOL to the time parents reported trying to fall asleep. Diary-reported Sleep Offset, number of awakenings, WASO, SSQ, and restedness were used directly without further derivation. Diary data were mainly used to guide the actigraphy algorithm (see below), and are only used as secondary outcome markers.

#### Actigraphy

Actigraphy processing was carried out using the Python package pyActigraphy in a Python v3.9.21 environment^101^. First, periods of subjectively reported non-wear were marked and excluded from all analyses. In addition, daytime non-wear periods were identified algorithmically using a modified version of the Choi et al. algorithm^102^. This algorithm scans the data for prolonged periods of inactivity and classifies intervals as non-wear when at least 150 consecutive minutes of zero activity counts are detected, while allowing up to two epochs with non-zero counts. In the present study, this algorithm was applied only during daytime intervals, defined as the period between 1 h after sleep offset and 1 h before the subsequent sleep onset based on sleep diaries. To reduce the influence of extreme artefacts, any 30-s epoch with an activity count exceeding four standard deviations above the group mean was replaced by the mean activity count of the surrounding 10-min window (affecting <1% of all data).

Next, epoch-by-epoch sleep–wake classification was performed using the Cole-Kripke algorithm with Webster’s rescoring rules applied^103^. Because the Cole-Kripke algorithm operates on 60-s epochs, the original 30-s data were first aggregated by taking the maximum count of each pair of consecutive epochs, following the original implementation by Cole et al.^103^. A wake threshold of one activity count was selected to maximize agreement with diary-reported sleep.

Sleep periods were then identified as sequences of at least ten consecutive epochs classified as “sleep,” allowing interruptions of up to five epochs scored as “wake”^104^. To ensure consistency with participant-reported sleep behavior, algorithm-derived sleep onset and offset times were subsequently compared against diary-reported sleep onset and offset. Only sleep periods falling within a 1-h window around the reported sleep interval were retained. When no sleep diary was available for a given day, the participant’s average sleep onset and offset across the study period were used as reference points.

Following sleep detection, standard rest-activity metrics were derived from the identified sleep periods. These included sleep onset, sleep offset, midsleep timing, time in bed (TIB; total duration of detected sleep intervals), TST (sum of all epochs scored as sleep within a sleep interval), sleep efficiency (TST/TIB), and sleep fragmentation (percentage of wake bouts occurring during the sleep interval). Variability in sleep patterns within and between parents was quantified using intraclass correlation coefficients (ICCs). Stability of the rest-activity rhythm was further characterized using the nonparametric measures Intradaily Variability (IV), Interdaily Stability (IS), and the SRI. Daytime rest was quantified as sleep episodes detected during daytime periods.

These actigraphy-derived metrics constituted the primary descriptive outcomes of the study. Unless explicitly stated otherwise, all reported sleep outcomes refer to actigraphy-derived measures rather than diary reports. To assess potential systematic bias between subjective and objective measures, Bland-Altman analyses were conducted comparing diary-reported and actigraphy-derived estimates of sleep onset, sleep offset, and WASO.

#### Light data

Using current weights provided by the Condor research team (on 02.12.2024), we computed the photopic illuminance and the melanopic equivalent daylight illuminances (melanopic EDI) derived from the Actlumus devices. The recorded photopic illuminance and melanopic EDI were log-10 transformed. Self-reported non-wear periods were matched with light data and removed. The light exposure during sleep episodes was obtained from the wrist-worn Actiwatch and log-10 transformed.

Descriptive statistics of parents’ light exposure patterns included the averaged photopic and melanopic exposure over 24-h days—and over the waking and sleeping hours for each of the observation days—and were derived with the LightLogR package^105^. Photoperiods were computed according to civil dawn and dusk. Wake and sleep episodes were determined using actigraphy-derived sleep onset and sleep offset. If sleep onset and sleep offset could not be derived from actigraphy, sleep diary entries were used. Time (TaT), Mean- (MLiT), First- and Last Lighting time above threshold were assessed at thresholds: 10 lux and 50 lux (during sleep episode), 100 lux, 250 lux, 1000 lux, and 10,000 lux. The Interdaily Stability (IS) and Intradaily Variability (IV) of the light exposure were estimated over the entire 2-week sampling period. Variance in these light metrics within and between parents was explored with ICCs. Unless mentioned otherwise, light-derived metrics were computed on photopic illuminance.

### Thematic stratification

Following preprocessing and variable derivation, we organized our descriptive data into a set of themes that reflect key dimensions of light and sleep ecologies among parents, as well as the potential impact of gender and infant characteristics. Themes were selected based on both theoretical relevance and patterns emerging from the raw data, and are presented in Table 5. Within each theme, we computed descriptive summary statistics (mean, standard deviation, range) for all quantitative measures related to that theme (e.g., TST, sleep efficiency, IS, IV, photopic illuminance, and melanopic EDI). To present intra-individual variability in a theme, we selected representative case profiles and displayed and discussed their individual light exposure and/or rest-activity patterns.

**Table 5:** Themes organized by key categories

Key	Theme
Sleep	Nighttime sleep: adequate duration with frequent disruptions
	From consolidated to fragmented: individual differences in parental sleep
	Subjective sleep quality does not reflect objective sleep
	Daytime napping behavior
	Stable days, variable nights
Light	To each their own light: interindividual differences in light exposure
	Traces of seasons (on days and nights)
	High variability of light exposure
Infant	How the baby can shape parental sleep and light exposure
Gender	Unequal responsibilities during the night
	Gender differences in daytime napping and light exposure

Given the descriptive and exploratory aims of the study, analyses focused on characterizing patterns, variability, and contextual differences in sleep and light exposure rather than on formal hypothesis testing. The present manuscript was intended as an initial descriptive overview of the dataset, aimed at transparently illustrating the substantial within- and between-parent variability in sleep and light ecologies during early parenthood. As such, no a priori hypotheses or inferential statistical analyses were specified for the current paper. Instead, we provide a mixed-methods description of sleep–wake and light exposure patterns to generate hypotheses and identify potentially relevant associations that can be examined more formally in subsequent hypothesis-driven studies using this dataset.

## Supplementary information

Supplementary Information

## Data Availability

The datasets generated and/or analyzed during the current study are not publicly available due to privacy and ethical restrictions. Pseudo-anonymised datasets can be made available from the corresponding author on reasonable request for academic and non-commercial research purposes.
