# Supplementary material for: Sleep and light ecologies of parents with young infants: descriptive findings from the SUNSHINE project
Source: NPJ Biol Timing Sleep. 2026 Jul 27;3:37. doi: 10.1038/s44323-026-00098-w (PMC13408594; doi:10.1038/s44323-026-00098-w)
Supplement: Supplementary file 1 — Supplementary Information [file 44323_2026_98_MOESM1_ESM.pdf]

SUPPLEMENTARY MATERIALS

Sleep and Light Ecologies of Parents with Young Infants: Descriptive Findings from the SUNSHINE Project

CONTENTS

S1. Questionnaires used in the study 2

1. Infant Sleep - Brief Infant Sleep Questionnaire–Revised 2
2. Parental Sleep Quality – Pittsburgh Sleep Quality Index 2
3. Chronotype – ultra-short Munich Chronotype Questionnaire 3
4. Depressive Symptoms – Edinburgh Postnatal Depression Scale 3
5. Daytime sleepiness – Epworth Sleepiness Scale 3
6. Parental Stress – Parental Stress Scale 4

S2. Supplementary Figures 5

S3. Supplementary Tables 12

S1. QUESTIONNAIRES USED IN THE STUDY

During the intake, participants completed a series of self-report questionnaires administered via LimeSurvey to assess demographics, infant sleep patterns and behaviours, parental sleep quality, chronotype, depressive symptoms, daytime sleepiness, and perceived stress. These included the Infant Sleep Questionnaire–Revised (BISQ–R;[?]), Pittsburgh Sleep Quality Index (PSQI;[?]), ultra-short Munich Chronotype Questionnaire (μMCTQ;[?]), Edinburgh Postnatal Depression Scale (EPDS;[?]), Epworth Sleepiness Scale (ESS;[?]), and Parental Stress Scale (PSS;[?]). Detailed descriptions of all questionnaires are provided below.

1. Infant Sleep - Brief Infant Sleep Questionnaire–Revised

Infant sleep during the previous two weeks was assessed using the *Brief Infant Sleep Questionnaire–Revised* (BISQ–R;[?]). The BISQ–R is a parent-report instrument for infants and toddlers up to three years of age. It provides estimates of sleep parameters, including sleep onset time, nocturnal and daytime sleep duration, total sleep duration, number and duration of nightly awakenings, sleep onset latency, and the longest continuous sleep period. In addition, it assesses contextual aspects of infant sleep, such as sleep arrangements (e.g., sleep location, bed- or room-sharing), sleep-related behaviors (e.g., nighttime feeding, soothing practices), and parental perception of the child's sleep as problematic. The original BISQ has demonstrated high test–retest reliability for core parameters ($r > .82$) and good validity compared with actigraphy and daily sleep diaries[?] , while subsequent studies have confirmed the BISQ–R's broader content validity and cross-cultural applicability[?,?] .

2. Parental Sleep Quality – Pittsburgh Sleep Quality Index

Parental sleep quality during the past month was measured with the *Pittsburgh Sleep Quality Index* (PSQI;[?]). The PSQI comprises 19 self-rated items that yield seven component scores (sleep quality, sleep latency, sleep duration, habitual sleep efficiency, sleep disturbances, use of sleep medication, and daytime dysfunction) and a global score ranging from 0 to 21, with scores >5 indicating clinically relevant sleep disturbance. The PSQI has shown good internal consistency (Cronbach's $\alpha \approx .83$), strong test–retest reliability, and robust construct validity across both clinical and community samples[?,?] . In the present

sample, internal consistency of the 19 items was low (Cronbach's $\alpha = .42$).

3. *Chronotype – ultra-short Munich Chronotype Questionnaire*

Current chronotype was assessed using the *ultra-short Munich Chronotype Questionnaire* (μMCTQ;[?]). The μMCTQ derives chronotype from self-reported sleep–wake timing on workdays and free days, producing a corrected mid-sleep point on free days (MSFsc) as its main outcome. Based on MSFsc, chronotypes can be categorized into early ($\leq 3.5\,\text{h}$), intermediate ($> 3.5 - 5.5\,\text{h}$), and late ($\geq 5.5\,\text{h}$) types, reflecting established population-based cutoffs[?] . The μMCTQ has demonstrated good external validity, as MSFsc aligns closely with objective sleep timing (actigraphic mid-sleep; $r \approx 0.7$) and shows a moderate–strong association with circadian phase as indexed by dim-light melatonin onset (up to $r \approx 0.68$ in adults, though estimates vary by cohort and method)[?, ?, ?, ?] .

4. *Depressive Symptoms – Edinburgh Postnatal Depression Scale*

Depressive symptoms during the previous seven days were assessed with the *Edinburgh Postnatal Depression Scale* (EPDS;[?]). The EPDS is a 10-item self-report questionnaire widely used for detecting perinatal depressive symptoms. Items assess mood, guilt, anxiety, anhedonia, sleep disturbance, and suicidal ideation, with total scores ranging from 0 to 30. A score $\geq$10 indicates possible depression, and $\geq$13 is commonly used as a cut-off for probable major depression. The EPDS has demonstrated good internal consistency (Cronbach's $\alpha = .80$–.87 as reported in literature, Cronbach's $\alpha = .72$ in our sample), strong test–retest reliability, and solid construct validity across diverse perinatal populations[?, ?, ?] .

5. *Daytime sleepiness – Epworth Sleepiness Scale*

Current daytime sleepiness was measured with the *Epworth Sleepiness Scale* (ESS;[?]). The ESS consists of 8 items assessing the likelihood of dozing in common situations (e.g., watching television, reading, sitting in a meeting). Scores range from 0 to 24, with scores $\geq$10 indicating excessive daytime sleepiness. The ESS demonstrated good internal consistency in the present sample (Cronbach's $\alpha = .83$), comparable to previously reported val-

ues ($\alpha \approx .73$–$.88$) and consistent with its established reliability and validity across diverse populations[?][?][?].

6. *Parental Stress – Parental Stress Scale*

Perceived parental stress was measured with the *Parental Stress Scale* (PSS;[?]), an 18-item questionnaire capturing both positive aspects of parenting (e.g., emotional benefits, personal fulfillment) and negative aspects (e.g., demands, restrictions). Total scores range from 18 to 90, with higher scores indicating greater stress. In the present sample, the PSS showed acceptable internal consistency (Cronbach's $\alpha = .76$), comparable to previously reported reliability estimates ($\alpha \approx .80$)[?].

S2. SUPPLEMENTARY FIGURES

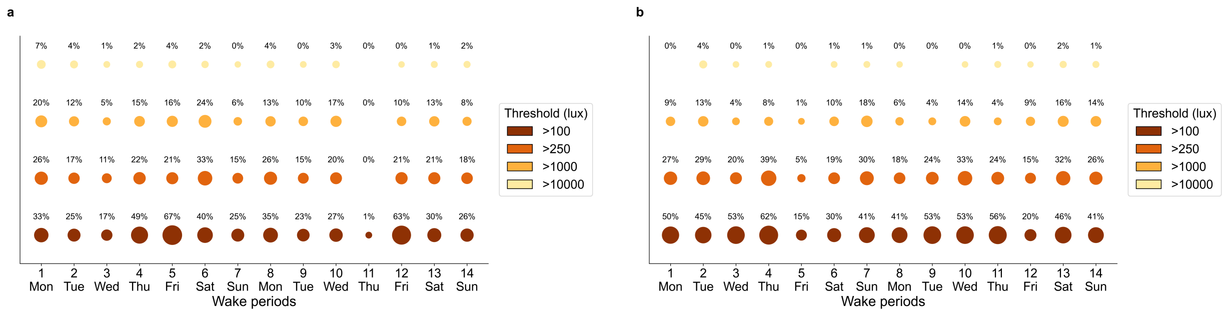

FIG. S1. **Time above thresholds during wake episodes of (a) parent F-201 ("fragmented sleeper," July–August 2024) and (b) parent F-271 ("pristine sleeper," late September 2024). Parent F-201 (a) spent short durations at light levels above 250 lux during wake, despite the summer time, with irregular duration above thresholds between days. On the other side, parent F-271 (b) had long exposures at lower light levels (100 and 250 lux thresholds) yet had very short (to none) exposure duration at high light levels (1,000 and 10,000 lux). By comparison, parent F-271' light exposure pattern appears more regular between days.** *Note:* Time above each threshold is expressed as a percentage of time awake.

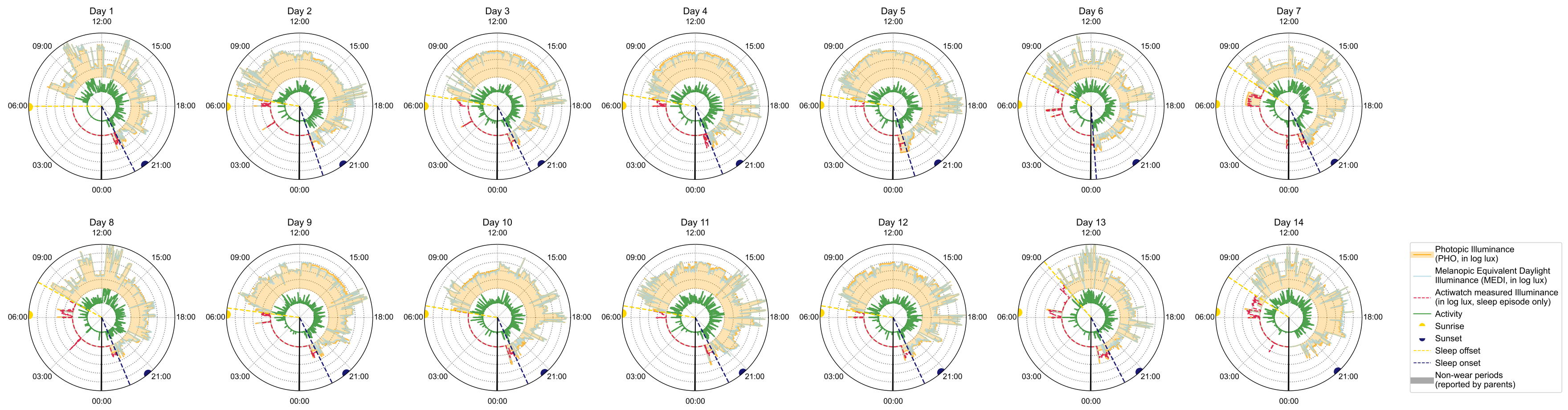

FIG. S2. Light exposure of parent F-231-C1 over the two weeks (starting on Monday) showing both "stable days" and "dynamic days" in light exposure. On Day 2 to 5 (Tuesday to Friday), peaks of light could be seen in the early morning and late afternoon, but in between, the light pattern appeared smooth, devoid of any large transitions between lighting levels. We defined these days as “stable days". On the contrary, light on Days 1, 6, 7 (Monday, Saturday, and Sunday) appeared to fluctuate more frequently from dimmer to brighter levels during the day, giving the sense of “dynamic days". This pattern was also noticeable the following week, with days appearing again more stable from Tuesday to Friday (Day 9 to 12) and dynamic days the rest of the week (Day 8, 13, 14).

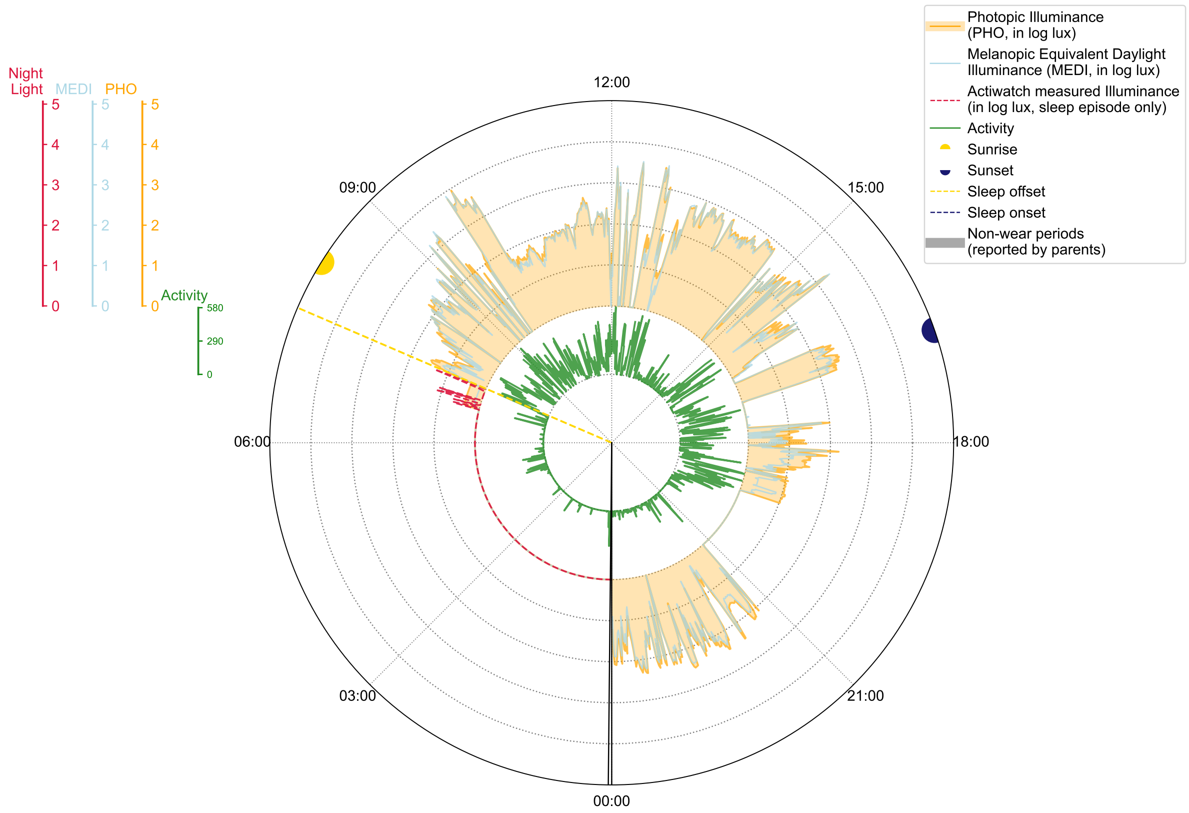

FIG. S3. Light exposure of parent F-301-C3 on day 5. Light appears constant (stable) between 10:00–12:00 and 13:00–15:00, and fluctuates more outside these times. Note that the stable light episode in the late morning occurred simultaneously with high wrist activity.

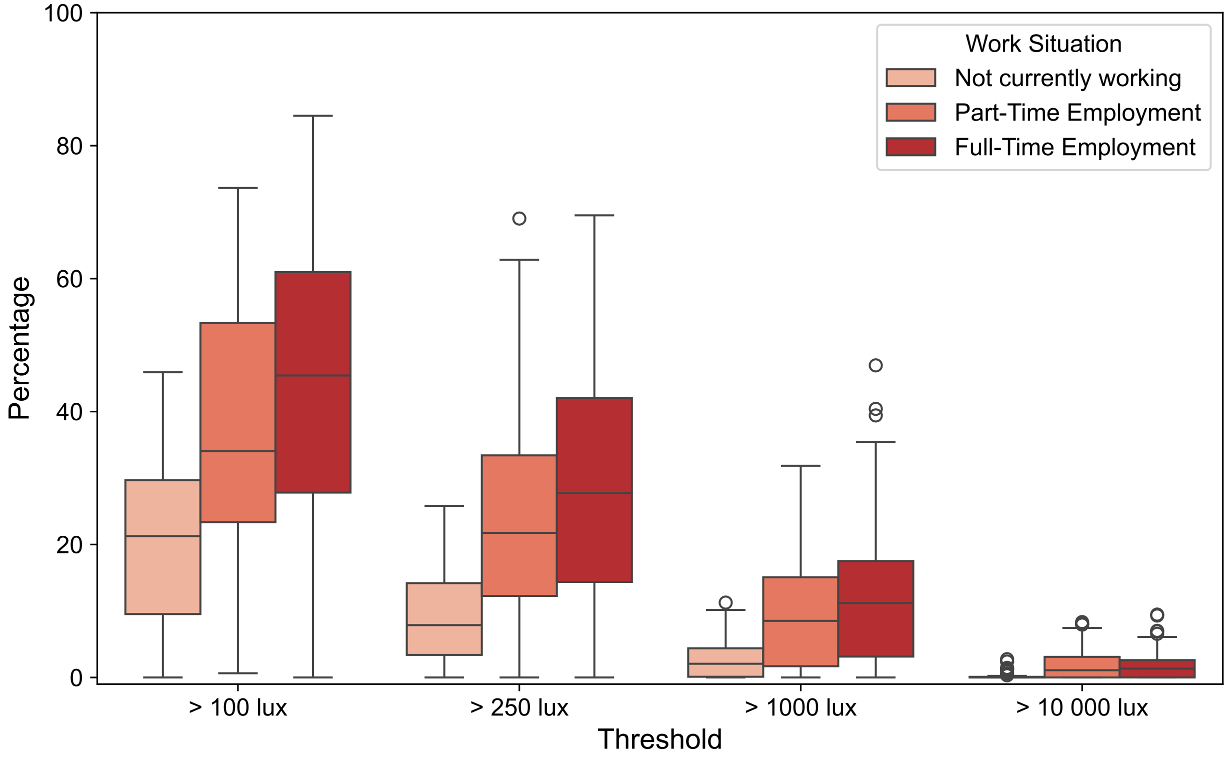

FIG. S4. Average time above light threshold for full-time working, part-time working, and not-working parents, expressed as percentage of time awake. Within the sub-sample of parents working full or part-time jobs, we observed that work-days appeared to correspond to days when light appeared more stable, and work-free days with more dynamic light. On average, it seems that full-time working parents spent more wake time above 100, 250, or 1000 lux thresholds, than part-time working parents or not-working parents.

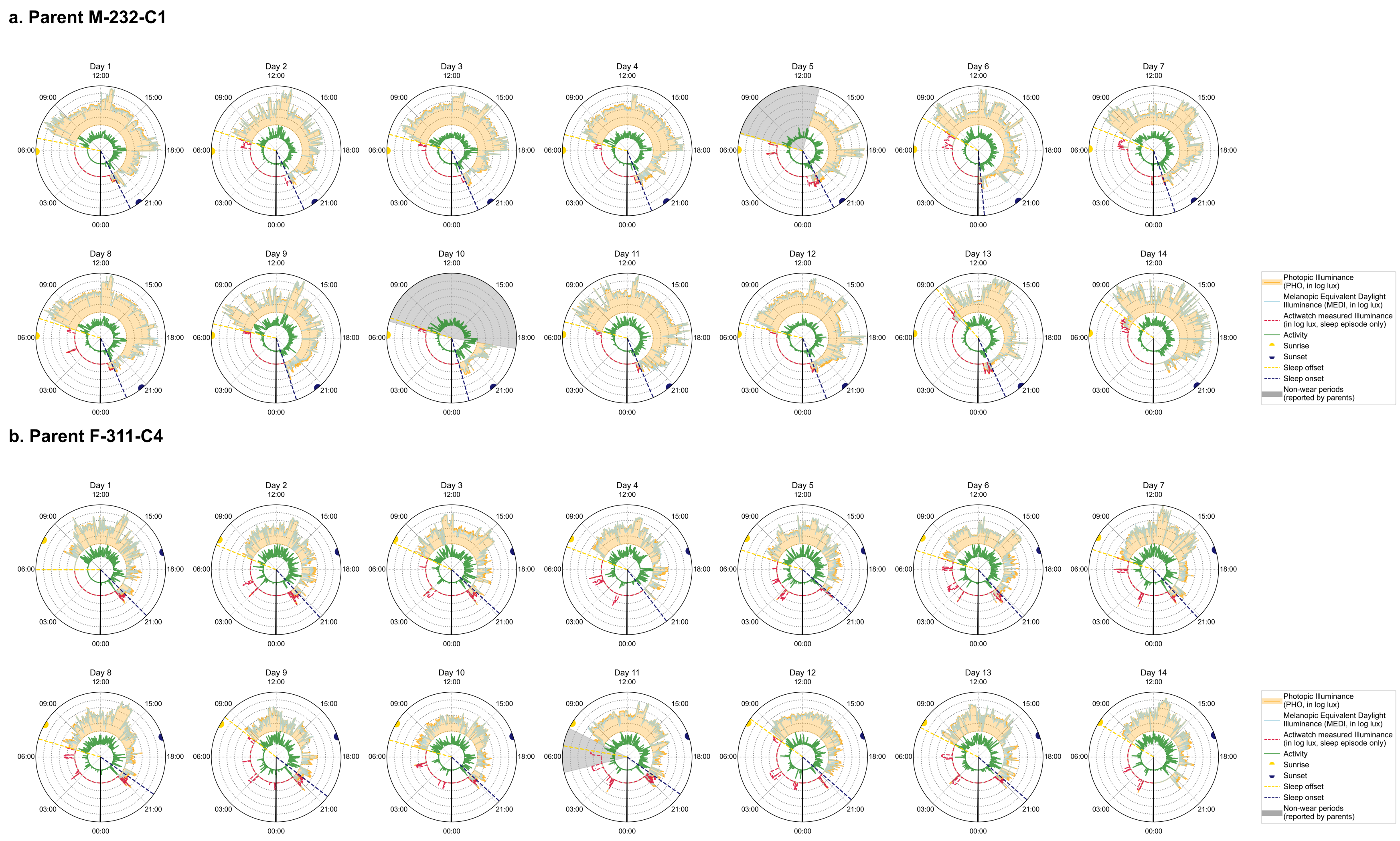

FIG. S5. Light exposure of parent M-232-C1 *(a)* and parent F-311-C4 *(b)* over two weeks, starting on Monday. High illuminance (peak of light) can be seen around 12:00 to 13:00, reaching above 1000 lux on most of Parent M-232-C1's days (a), but more specifically on Day 1-4, 6 and 8. For parent F-311-C4 (b), periods of darkness (dips of light) can be observed almost every day, starting between 19:00 and 20:00, for a duration of 30 to 60 minutes. For parent F-311-C4, these short light interruptions or dips corresponded to the usual bedtime of their infant (19:30).

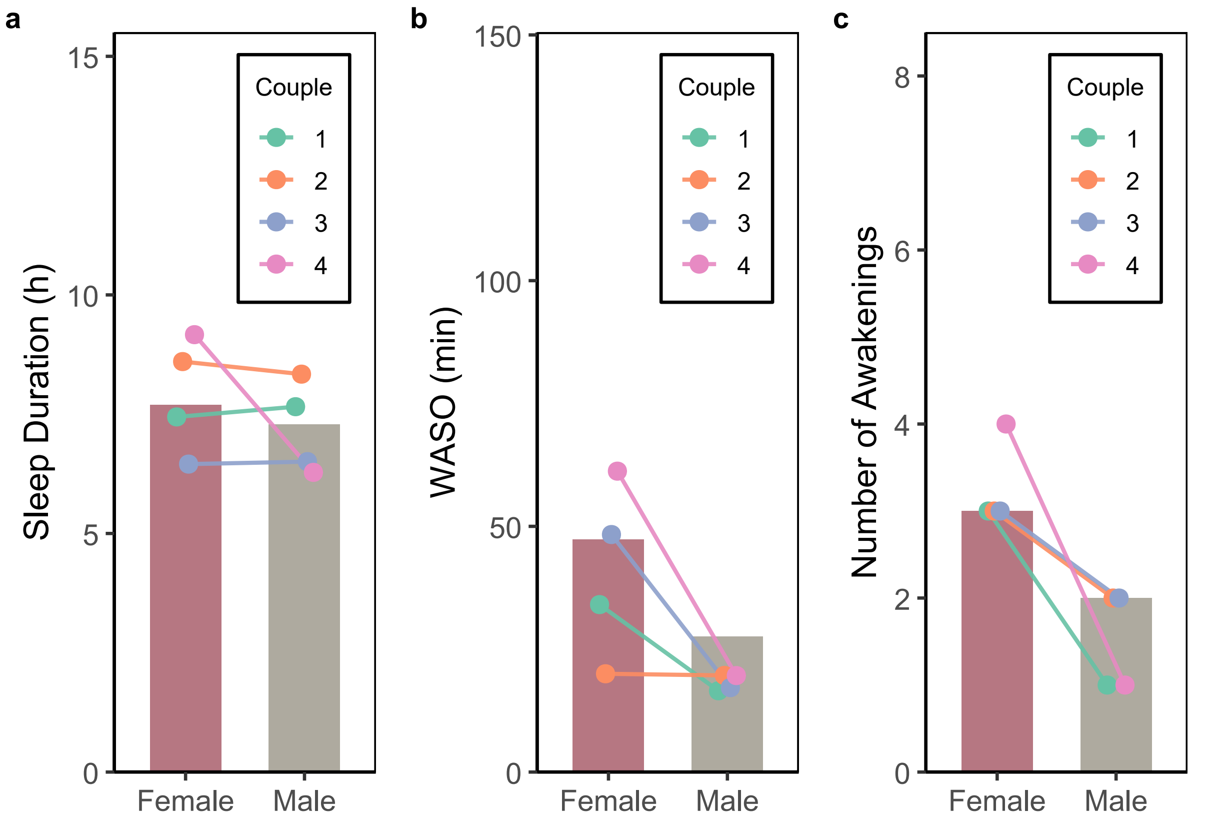

FIG. S6. **Differences in sleep parameters between mothers and fathers, with within-dyad comparisons.** Panels show *(a)* mean Sleep Duration, *(b)* mean Wake After Sleep Onset (WASO), and *(c)* median Number of Awakenings. Bars denote the group-level statistics for female (deep rd) and male (olive) parents, while points and connecting lines trace each mixed-gender couple's values (with a small horizontal jitter).

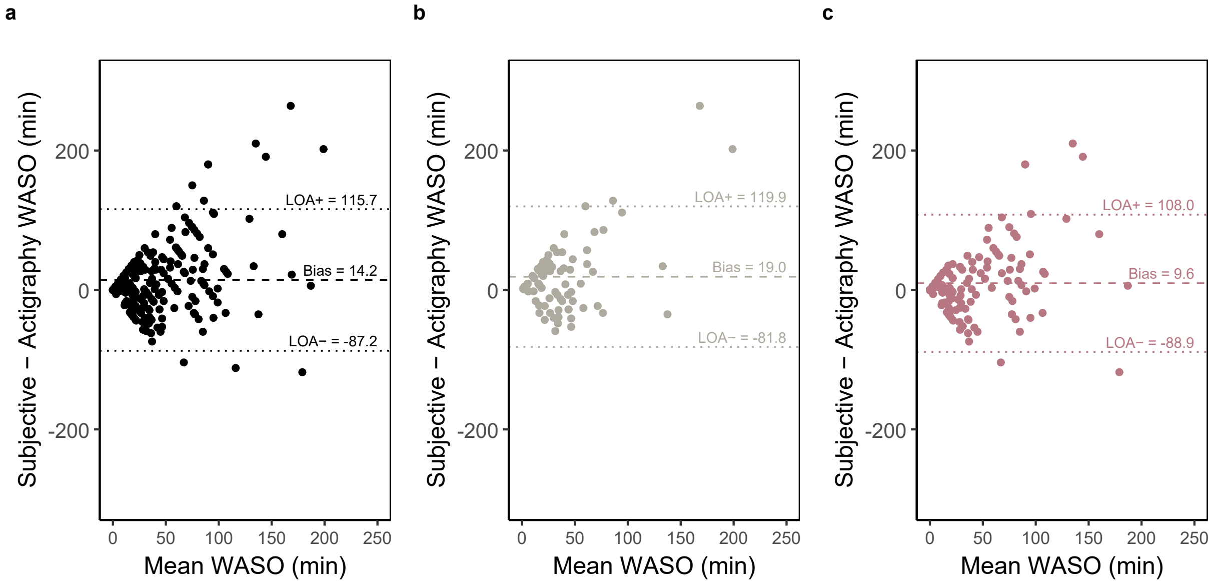

FIG. S7. **Bland–Altman comparison of diary versus actigraphy wake-after-sleep-onset (WASO).** Panels show the difference between subjective (diary) and objective (actigraphy) WASO plotted against their mean, with per-panel bias (dashed line) and 95% limits of agreement (dotted lines; LOA) annotated. *(a)* All parents (N = 166 nights) over-estimate WASO by a mean of +14.2 min (LOA –87.2 to +115.7 min). *(b)* Fathers (N = 76 nights) over-estimate by +19.0 min (LOA –81.8 to +119.9 min). *(c)* Mothers (N = 90 nights) over-estimate by +9.6 min (LOA –88.9 to +108.0 min). In all panels the cloud "fans out" at higher mean WASO, indicating increasing variability in recall accuracy on more fragmented nights.

S3. SUPPLEMENTARY TABLES

TABLE S1. Average sleep per parent and group average

Parent	Onset (HH:MM)	Offset (HH:MM)	Midsleep (HH:MM)	TIB (HH:MM)	Nr. Awakenings	WASO (HH:MM)	TST (HH:MM)	Longest stretch (HH:MM)	Efficiency (%)	Fragmentation (%)	Sleep Quality (1–7)	Restedness (1–7)
**F-201**	21:42 (± 01:06)	07:37 (± 00:30)	02:40 (± 00:33)	09:53 (± 01:22)	3.38 (± 1.94)	01:31 (± 01:06)	08:22 (± 01:19)	04:24 (± 01:13)	85.1 (± 10.8)	22.1 (± 10.6)	5.0 (1.0–6.0)	3.0 (1.0–6.0)
**S24211**	21:36 (± 00:31)	07:20 (± 00:31)	02:28 (± 00:27)	09:44 (± 00:31)	1.67 (± 1.61)	00:38 (± 00:52)	09:05 (± 00:57)	07:35 (± 02:16)	93.4 (± 8.8)	11.0 (± 10.6)	4.0 (1.0–6.0)	3.0 (2.0–6.0)
**M-221**	22:43 (± 00:50)	07:22 (± 00:49)	03:03 (± 00:34)	08:39 (± 01:16)	3.46 (± 1.45)	00:31 (± 00:23)	08:07 (± 01:05)	04:18 (± 01:13)	94.1 (± 4.0)	15.0 (± 4.2)	4.0 (1.0–6.0)	3.0 (1.0–3.0)
**F-231-C1**	22:28 (± 00:24)	07:15 (± 00:53)	02:52 (± 00:32)	08:46 (± 00:55)	3.46 (± 1.27)	00:34 (± 00:30)	08:12 (± 00:46)	05:02 (± 01:44)	93.8 (± 5.2)	14.7 (± 4.0)	4.0 (2.0–6.0)	4.0 (3.0–6.0)
**M-232-C1**	22:33 (± 00:23)	07:24 (± 00:41)	02:59 (± 00:22)	08:51 (± 00:51)	0.92 (± 0.76)	00:16 (± 00:26)	08:35 (± 00:49)	08:04 (± 01:06)	97.0 (± 4.9)	7.4 (± 4.2)	5.0 (2.0–7.0)	5.0 (3.0–6.0)
**F-241-C2**	20:54 (± 00:25)	07:12 (± 00:34)	02:03 (± 00:24)	10:18 (± 00:36)	2.62 (± 1.19)	00:20 (± 00:14)	09:58 (± 00:38)	07:03 (± 02:24)	96.8 (± 2.4)	11.4 (± 3.3)	2.5 (2.0–5.0)	3.0 (2.0–5.0)
**M-242-C2**	21:13 (± 00:25)	06:58 (± 00:34)	02:06 (± 00:17)	09:45 (± 00:51)	1.50 (± 0.90)	00:19 (± 00:20)	09:25 (± 00:46)	07:34 (± 01:41)	96.7 (± 3.4)	7.9 (± 3.6)	3.0 (3.0–6.0)	3.0 (3.0–6.0)
**M-251**	22:05 (± 01:02)	07:19 (± 00:33)	02:42 (± 00:36)	09:13 (± 01:13)	2.00 (± 1.00)	00:20 (± 00:17)	08:52 (± 01:04)	05:10 (± 01:26)	96.5 (± 3.0)	9.3 (± 3.9)	4.0 (2.0–7.0)	3.0 (2.0–5.0)
**F-261**	22:23 (± 00:46)	07:07 (± 00:23)	02:45 (± 00:21)	08:44 (± 01:02)	1.00 (± 0.71)	00:16 (± 00:21)	08:28 (± 00:51)	07:40 (± 01:12)	97.2 (± 3.6)	6.5 (± 3.3)	6.0 (3.0–7.0)	5.0 (2.0–6.0)
**F-271**	23:38 (± 00:55)	07:59 (± 00:38)	03:49 (± 00:37)	08:19 (± 01:01)	0.69 (± 1.03)	00:11 (± 00:21)	08:07 (± 00:54)	07:18 (± 01:21)	97.8 (± 3.8)	5.9 (± 4.8)	6.0 (3.0–7.0)	5.0 (2.0–7.0)
**F-281**	21:13 (± 00:40)	06:34 (± 00:24)	01:53 (± 00:21)	09:20 (± 00:53)	1.31 (± 1.03)	00:22 (± 00:22)	08:57 (± 00:52)	08:12 (± 01:13)	96.0 (± 4.1)	7.5 (± 4.0)	6.0 (2.0–7.0)	4.0 (3.0–7.0)
**F-291**	21:44 (± 00:35)	06:47 (± 00:26)	02:15 (± 00:20)	09:02 (± 00:50)	3.77 (± 1.69)	00:49 (± 00:27)	08:13 (± 00:47)	03:46 (± 01:42)	90.9 (± 4.8)	16.2 (± 5.5)	4.0 (3.0–6.0)	4.0 (3.0–6.0)
**F-301-C3**	23:34 (± 00:38)	07:18 (± 00:26)	03:26 (± 00:17)	07:43 (± 00:57)	3.00 (± 1.15)	00:44 (± 00:40)	06:59 (± 00:50)	04:04 (± 01:14)	90.8 (± 7.8)	17.7 (± 7.9)	3.0 (1.0–4.0)	2.0 (1.0–3.0)
**M-302-C3**	23:34 (± 00:24)	07:18 (± 00:27)	03:26 (± 00:20)	07:44 (± 00:34)	1.77 (± 0.93)	00:15 (± 00:25)	07:29 (± 00:37)	05:17 (± 01:28)	96.8 (± 5.2)	8.7 (± 4.8)	3.5 (2.0–5.0)	3.0 (2.0–5.0)
**F-311-C4**	20:51 (± 00:19)	07:30 (± 00:32)	02:11 (± 00:13)	10:39 (± 00:47)	4.15 (± 1.21)	01:01 (± 00:23)	09:38 (± 00:43)	04:20 (± 00:49)	90.5 (± 3.4)	16.5 (± 3.1)	4.5 (2.0–6.0)	4.0 (1.0–5.0)
**M-312-C4**	21:54 (± 00:34)	06:03 (± 00:46)	01:59 (± 00:19)	08:10 (± 01:14)	1.54 (± 1.27)	00:21 (± 00:18)	07:48 (± 01:05)	06:06 (± 01:41)	95.9 (± 3.5)	8.3 (± 4.9)	3.0 (2.0–6.0)	4.0 (2.0–6.0)
**M-321**	22:45 (± 01:39)	08:03 (± 00:38)	03:27 (± 00:45)	09:10 (± 02:12)	3.54 (± 1.71)	01:04 (± 00:38)	08:06 (± 01:59)	05:14 (± 01:58)	87.7 (± 7.2)	18.4 (± 7.0)	4.5 (1.0–6.0)	3.0 (2.0–5.0)
**F-331**	22:18 (± 01:01)	08:20 (± 00:41)	03:19 (± 00:38)	10:00 (± 01:13)	4.23 (± 1.79)	01:12 (± 00:52)	08:47 (± 01:12)	04:22 (± 02:19)	88.1 (± 7.9)	19.2 (± 9.3)	2.0 (1.0–5.0)	2.0 (1.0–4.0)
**F-341**	00:23 (± 02:28)	09:20 (± 01:27)	04:34 (± 01:51)	09:54 (± 03:36)	3.69 (± 1.97)	01:51 (± 03:45)	08:02 (± 01:38)	04:52 (± 01:25)	86.6 (± 17.8)	19.8 (± 15.2)	4.5 (2.0–5.0)	3.0 (1.0–6.0)
**Group Average**	21:02 (± 00:48)	07:25 (± 00:37)	02:50 (± 00:31)	09:09 (± 01:09)	2.51 (± 1.30)	00:40 (± 00:40)	08:29 (± 01:00)	05:48 (± 01:33)	93.2 (± 5.9)	12.8 (± 6.0)	4.0 (1.0–7.0)	3.0 (1.0–7.0)

Note. Data are represented as mean (± SD) or median (range) and are based on diary-reported sleep episodes. † TIB: Time in Bed; WASO: Wake After Sleep Onset; TST: Total Sleep Time.

TABLE S2. Bland–Altman agreement between diary-reported and actigraphy-derived sleep estimates

Measure	n	Bias (min)	SD diff. (min)	95% LOA (min)	Slope	p
**Sleep onset**	206	48.3	17.6	[13.8, 82.9]	0.006	.029
**Sleep offset**	206	-29.6	21.6	[-71.8, 12.6]	0.049	.058
**WASO**	206	12.6	37.6	[-61.1, 86.2]	0.348	$<$ .001

Note. Bias is diary minus actigraphy. LOA = limits of agreement. For sleep onset and sleep offset, differences are wrapped circular differences in minutes.

TABLE S3. Average light exposure per parent during waking periods

	MEDI			PHO			TAT>100 lux				TAT>250 lux				TAT>1000 lux				TAT>10000 lux			
Id	mean	std	range	mean	std	range	Dur. (%)	Av. Time	First	Last	Dur. (%)	Av. Time	First	Last	Dur. (%)	Av. Time	First	Last	Dur. (%)	Av. Time	First	Last
**F-201**	1.44	1.18	0.00 - 4.97	1.57	1.17	0.00 - 5.00	33.51	15:00:29	09:34:08	19:12:38	19.53	15:05:06	09:33:36	18:49:50	12.50	15:10:58	10:19:32	18:40:00	2.54	14:50:04	12:36:27	16:51:00
**M-221**	1.85	0.96	0.00 - 4.96	2.01	0.91	0.00 - 5.03	58.11	13:37:55	07:58:38	20:56:42	34.95	13:31:09	08:06:00	18:48:42	10.40	14:32:39	08:58:00	18:09:47	1.84	14:45:06	12:06:25	16:22:04
**F-231-C1**	1.96	1.05	0.00 - 5.02	2.08	1.02	0.00 - 5.06	51.83	13:18:45	07:37:25	20:16:25	41.47	13:08:25	07:43:04	19:47:21	15.90	13:07:52	08:25:51	17:51:17	2.85	14:48:49	11:59:12	16:17:08
**M-232-C1**	1.95	1.10	0.00 - 4.96	2.07	1.06	0.00 - 5.00	44.67	13:51:20	08:54:47	20:23:38	38.19	13:40:27	08:56:51	19:25:42	15.85	13:04:57	08:45:13	18:20:46	3.91	13:09:14	09:50:32	16:31:09
**F-241-C2**	1.62	1.20	0.00 - 4.98	1.68	1.20	0.00 - 5.03	45.19	13:58:39	07:43:18	20:03:18	30.35	14:13:31	08:26:36	19:49:32	14.04	15:18:46	09:50:50	19:27:36	1.80	15:59:22	13:16:45	18:11:35
**M-242-C2**	2.02	1.18	0.00 - 4.89	2.11	1.14	0.00 - 4.95	58.33	14:05:31	07:46:21	20:31:00	44.06	14:11:58	08:19:08	20:10:47	23.26	14:58:59	09:01:17	19:56:51	3.30	15:31:39	12:16:12	18:14:51
**M-251**	2.18	1.06	0.00 - 5.00	2.29	1.02	0.00 - 5.03	64.96	14:08:56	07:56:04	20:45:34	49.85	14:08:40	08:09:25	19:57:04	21.78	14:27:36	08:43:21	18:41:04	3.79	14:22:16	11:32:00	16:36:38
**F-261**	1.80	1.07	0.00 - 4.94	1.91	1.06	0.00 - 4.98	51.48	13:27:19	07:57:38	19:29:04	32.69	13:27:20	08:08:55	18:35:34	12.23	13:48:53	08:33:21	18:03:38	2.55	14:34:03	11:53:49	16:40:10
**F-271**	1.59	1.05	0.00 - 4.85	1.73	1.03	0.00 - 4.91	43.83	14:19:12	08:24:21	17:57:38	24.89	13:49:28	08:40:30	20:44:42	9.65	12:58:53	09:22:08	17:40:04	1.12	12:10:15	10:32:32	15:20:21
**F-281**	1.18	0.87	0.00 - 4.74	1.39	0.86	0.00 - 4.79	18.31	13:00:11	08:56:55	19:09:38	9.23	12:56:22	10:39:21	15:20:42	3.76	12:47:25	10:46:54	14:36:38	0.62	12:42:44	10:20:45	14:47:45
**F-291**	0.90	0.74	0.00 - 3.55	1.08	0.81	0.00 - 3.59	13.93	12:36:57	07:54:25	18:19:08	5.39	12:43:08	10:50:30	14:56:00	0.04	13:14:15	13:09:30	13:23:00	0.00			
**F-301-C3**	1.17	0.90	0.00 - 4.62	1.36	0.91	0.00 - 4.71	27.21	13:56:32	08:58:17	21:38:42	11.59	13:08:39	09:51:13	19:04:55	2.16	12:14:57	10:57:49	13:54:27	0.20	11:31:21	11:02:12	11:56:12
**M-302-C3**	1.20	0.87	0.00 - 4.62	1.40	0.87	0.00 - 4.75	22.19	14:00:05	08:32:13	21:02:09	9.82	12:30:21	09:19:46	17:40:46	2.59	12:32:50	10:52:54	13:54:48	0.23	12:06:28	11:05:12	12:57:12
**F-311-C4**	1.30	0.96	0.00 - 4.91	1.44	0.94	0.00 - 4.99	27.21	12:29:36	08:59:47	17:28:04	12.89	12:47:34	09:47:17	15:27:08	5.15	13:08:36	11:33:35	14:12:20	0.53	13:13:59	12:25:24	13:53:48
**M-312-C4**	1.28	0.99	0.00 - 4.63	1.43	0.98	0.00 - 4.68	30.48	13:08:43	07:50:04	18:10:30	15.58	13:08:07	08:52:55	16:22:42	4.18	13:06:12	11:35:15	14:06:10	0.23	11:48:35	11:29:40	12:53:00
**M-321**	1.38	0.97	0.00 - 4.67	1.55	0.93	0.00 - 4.76	33.15	13:02:44	08:49:55	19:32:51	18.59	12:56:33	09:31:00	17:02:47	5.30	12:32:59	10:25:05	14:34:25	0.86	11:33:26	10:11:51	12:43:00
**F-331**	0.38	0.53	0.00 - 2.61	0.51	0.66	0.00 - 2.74	0.69	12:43:43	10:47:22	13:27:07	0.12	13:33:26	13:25:20	13:36:10	0.00				0.00			
**F-341**	0.85	0.71	0.00 - 4.35	1.06	0.74	0.00 - 4.44	6.96	15:09:36	11:02:32	17:09:09	3.10	14:10:42	13:06:45	15:58:15	1.04	14:09:56	13:49:48	14:37:36	0.02	12:57:30	12:57:00	12:58:00

Note. mean and standard deviation (std) are in log lux. Time above threshold (TAT) durations (Dur.) are presented as percentage over time awake.

TABLE S4. Average light exposure per parent during sleeping periods

	PHO			TAT>10 lux				TAT>50 lux				TAT>100 lux				TAT>250 lux			
Id	mean	std	Range	Dur. (%)	Av. Time	First	Last	Dur. (%)	Av. Time	First	Last	Dur. (%)	Av. Time	First	Last	Dur. (%)	Av. Time	First	Last
**F-201**	0.26	0.46	0.00 - 2.33	10.73	02:23:52	23:16:55	06:48:04	0.32	00:59:37	00:07:26	01:21:52	0.14	01:03:57	01:02:53	01:04:50	0.00			
**M-221**	0.06	0.25	0.00 - 2.26	2.33	04:08:51	01:01:30	06:46:42	0.11	05:31:30	05:30:40	05:32:55	0.02	08:40:30	08:40:00	08:41:00	0.00			
**F-231-C1**	0.13	0.36	0.00 - 1.92	7.43	01:40:15	22:30:03	07:05:36	0.12	02:21:31	00:52:59	02:54:44	0.00				0.00			
**M-232-C1**	0.12	0.33	0.00 - 2.34	3.86	04:13:20	23:50:58	07:07:45	0.06	08:07:35	08:04:00	08:14:00	0.03	09:05:39	08:58:59	09:18:00	0.00			
**F-241-C2**	0.14	0.39	0.00 - 2.76	4.58	05:09:50	02:33:32	06:52:55	1.68	06:03:52	05:37:49	06:40:25	0.83	05:50:36	05:21:09	06:36:22	0.15	06:53:02	06:50:19	06:56:00
**M-242-C2**	0.11	0.36	0.00 - 2.49	4.80	00:48:37	22:04:41	06:43:55	1.20	00:56:25	23:25:59	02:46:40	0.41	01:35:43	01:23:48	02:58:29	0.04	06:10:45	06:10:29	06:10:59
**M-251**	0.06	0.25	0.00 - 2.76	2.90	02:41:44	00:18:12	06:19:10	0.20	03:20:43	01:24:10	04:36:57	0.09	07:26:21	07:24:30	07:30:00	0.08	07:29:43	07:25:59	07:37:00
**F-261**	0.06	0.26	0.00 - 2.05	2.74	03:42:36	00:01:31	06:28:05	0.22	04:11:43	04:11:34	04:12:04	0.01	21:47:00	21:47:00	21:47:00	0.00			
**F-271**	0.03	0.19	0.00 - 2.33	1.46	05:27:12	05:25:24	06:33:34	0.24	01:02:42	00:56:38	01:09:46	0.09	01:05:25	00:59:55	01:08:26	0.00			
**F-281**	0.05	0.24	0.00 - 2.08	2.92	00:25:41	21:26:07	05:17:08	0.17	21:07:04	21:01:40	21:13:00	0.05	21:20:30	21:15:59	21:25:00	0.00			
**F-291**	0.05	0.24	0.00 - 2.55	2.53	00:54:02	23:13:06	04:56:48	0.35	21:59:32	21:54:35	22:06:42	0.13	22:08:32	22:07:44	22:09:14	0.03	22:23:30	22:22:59	22:24:00
**F-301-C3**	0.08	0.27	0.00 - 2.08	2.82	01:34:26	00:16:04	03:18:19	0.08	23:36:48	23:36:21	23:37:21	0.05	23:36:21	23:36:21	23:36:21	0.00			
**M-302-C3**	0.02	0.14	0.00 - 1.64	0.49	05:13:04	04:54:40	05:53:57	0.00				0.00				0.00			
**F-311-C4**	0.13	0.37	0.00 - 1.94	7.26	01:09:15	21:09:42	06:31:52	0.12	23:04:16	21:22:19	23:12:50	0.00				0.00			
**M-312-C4**	0.06	0.27	0.00 - 2.18	2.82	23:58:32	22:51:05	01:21:04	0.62	23:21:05	22:39:48	00:37:00	0.03	22:08:29	22:08:29	22:08:29	0.00			
**M-321**	0.14	0.39	0.00 - 2.63	9.99	05:31:26	00:48:38	07:51:50	0.67	07:11:15	06:45:16	07:30:46	0.26	06:35:09	06:33:56	07:37:19	0.04	04:55:20	02:59:30	08:43:30
**F-331**	0.15	0.38	0.00 - 2.28	6.23	02:35:52	23:28:59	07:35:14	0.11	06:34:15	06:31:59	06:36:29	0.10	08:54:30	08:50:00	08:58:59	0.00			
**F-341**	0.21	0.42	0.00 - 2.64	6.06	05:55:02	01:44:42	08:35:25	0.74	07:59:00	07:17:42	08:21:15	0.30	08:25:16	08:17:30	06:27:45	0.11	09:04:29	09:02:29	09:06:29

Note. mean and standard deviation (std) are in log lux. Time above threshold (TAT) durations (Dur.) are presented as percentage over time spent between sleep onset and sleep offset.

TABLE S5. Sleep metrics by gender.

**Metric**	**Fathers (n = 8)**	**Mothers (n = 11)**
Sleep Onset	22:24 (± 01:06)	22:11 (± 01:28)
Sleep Offset	07:13 (± 00:51)	07:26 (± 00:58)
Midsleep	02:49 (± 00:44)	02:47 (± 01:01)
Time In Bed	08:47 (± 01:23)	09:18 (± 01:36)
Number of Awakenings	2.11 (± 1.49)	2.62 (± 1.79)
Wake After Sleep Onset	00:27 (± 00:29)	00:45 (± 01:19)
Total Sleep Time	08:20 (± 01:16)	08:33 (± 01:14)
Longest Sleep Stretch	05:57 (± 01:57)	05:50 (± 02:13)
Sleep Efficiency	95.0 (± 5.5)	92.6 (± 8.6)
Fragmentation	10.8 (± 6.1)	13.6 (± 9.0)
Sleep Quality	4.0 (1.0–7.0)	5.0 (1.0–7.0)
Restedness	3.0 (1.0–6.0)	4.0 (1.0–7.0)
